# S, N‐Co‐Doped Graphene‐Nickel Cobalt Sulfide Aerogel: Improved Energy Storage and Electrocatalytic Performance

**DOI:** 10.1002/advs.201600214

**Published:** 2016-08-17

**Authors:** Guanjie He, Mo Qiao, Wenyao Li, Yao Lu, Tingting Zhao, Rujia Zou, Bo Li, Jawwad A. Darr, Junqing Hu, Maria‐Magdalena Titirici, Ivan P. Parkin

**Affiliations:** ^1^ Christopher Ingold LaboratoryDepartment of ChemistryUniversity College London20 Gordon StreetLondonWC1H 0AJUK; ^2^ School of Engineering and Materials Science/Materials Research InstituteQueen Mary University of LondonMile End RoadE14NSLondonUK; ^3^ State Key Laboratory for Modification of Chemical Fibers and Polymer MaterialsCollege of Materials Science and EngineeringDonghua UniversityShanghai201620China

**Keywords:** aerogel, nickel cobalt sulfide, oxygen reduction reaction, rechargeable alkaline battery

## Abstract

Metal sulfides are commonly used in energy storage and electrocatalysts due to their redox centers and active sites. Most literature reports show that their performance decreases significantly caused by oxidation in alkaline electrolyte during electrochemical testing. Herein, S and N co‐doped graphene‐based nickel cobalt sulfide aerogels are synthesized for use as rechargeable alkaline battery electrodes and oxygen reduction reaction (ORR) catalysts. Notably, this system shows improved cyclability due to the stabilization effect of the S and N co‐doped graphene aerogel (SNGA). This reduces the rate of oxidation and the decay of electronic conductivity of the metal sulfides materials in alkaline electrolyte, i.e., the capacity decrease of CoNi_2_S_4_/SNGA is 4.2% for 10 000 cycles in a three‐electrode test; the current retention of 88.6% for Co—S/SNGA after 12 000 s current–time chronoamperometric response in the ORR test is higher than corresponding Co—S nanoparticles and Co—S/non‐doped graphene aerogels. Importantly, the results here confirm that the Ni—Co—S ternary materials behave as an electrode for rechargeable alkaline batteries rather than supercapacitors electrodes in three‐electrode test as commonly described and accepted in the literature. Furthermore, formulas to evaluate the performance of hybrid battery devices are specified.

## 1 Introduction

Graphene has attracted great research interest after its first discovery in 2004.^1^ With 2D layered structure of carbon atoms, graphene possesses unique properties, such as high theoretical surface area (2630 m^2^ g^−1^),^2^ thermal and chemical stability,^3^ remarkable electronic and mechanical properties,^4^, ^5^ etc. Nevertheless, due to the strong van der Waals forces among the single carbon sheets, graphene has the tendancy to aggregate and form graphite,^6^ which leads to a sharp decrease in surface area and kinetic ion transport, affecting the performance of these materials when used in energy storage^4^ and electrocatalysis.^7^ In order to solve the aggregation problem and achieve fast ion and electron transfer, novel graphene structures have been developed. Reports include graphene foams prepared by template‐assisted chemical vapor deposition (CVD) methods,^8^ self‐assembly of aerosol or hydrogel by hydrothermal processes^9^, ^10^ or electrospray ionization,^11^ and layer‐by‐layer composite structures by filter assembly.^6^, ^12^ However, carbon materials alone, suffer from low charge storage capacity and limited active sites,^3^, ^13^, ^14^ which hinder their use as high‐performance energy storage devices and electrocatalysts.

One effective way to improve the performance of graphene materials, expand and improve their applications is to hybridize them with metal‐based semiconductors to form nanocomposites.^15^ Recently, nickel cobalt‐based oxides, sulfides, and selenides have been successfully used as the non‐noble metal cadidates in various electrochemical applications, such as Li‐ion batteries,^16^, ^17^ supercapacitors,^6^, ^18^, ^19^, ^20^, ^21^, ^22^ and eletrocatalysts.^23^, ^24^, ^25^ Various nanostructures have been designed and synthesized, such as nanosheets,^26^, ^27^ nanorods,^16^ self‐assembled nanoflowers,^18^ core–shell structures,^28^ mesoporous structures and dendritic structures,^29^ etc. For instance, Lou and co‐workers have fabricated nickel cobalt sulfide ball‐in‐ball hollow spheres via an anion exchange method and used them as supercapacitor electrodes,^28^ delivering a specific capacitance of 1036 F g^−1^ at a current density of 1 A g^−1^, and retaining 87% of its initial specific capacitance after 2000 cycles. Dai and co‐workers synthesized Co_1−_ *_x_*S/reduced graphene oxide hybrid catalysts showing a high oxygen reduction reaction (ORR) current density of 1.1 mA cm^−2^ at 0.7 V versus RHE with ≈100 μg cm^−2^ loading density.^30^ Compared with transitional metal oxides, transitional metal sulfides or selenides usually possess better electron conductivity.^31^, ^32^, ^33^ Taking into account their price, performance stabilities, and safety issues, metal sulfides are very attractive for electrochemical applications. The main hurdle to their use is their easy oxidization in alkaline electrolytes and the decay of electron conductivity especially during long‐term cycles.^34^ To address this problem, it is important to develop innovative Ni—Co—S nanostructures with improved stability and performances.

On the basis of the above idea, we designed and synthesized N, S co‐doped (S‐rich) graphene‐based nickel cobalt sulfide aerogels (Ni—Co—S/SNGA) with the aim to optimize the performances of metal sulfides, in particlar their cycling performance and illustrate their potential widespread application. The Ni—Co—S/SNGA exhibited improved electrochemical performance as electrodes for rechargeable alkaline batteries and as electrocatalysts for ORR in alkaline electrolyte.

## 2 Results and Discussion

**Figure 1:**
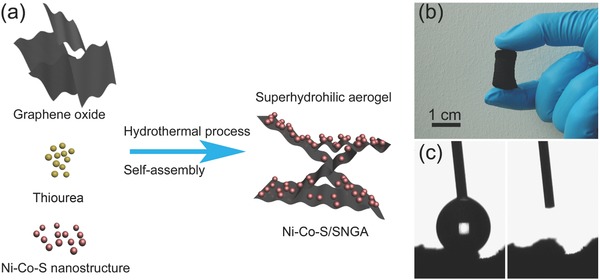
a) Schematic diagram of the synthesis of Ni—Co—S/SNGA; b) digital camera photo of the typical aerogel sample; and c) contact angle measurements before (left) and after (right) water dropping.

The main synthetic procedure to prepare Ni—Co—S/SNGA is illustrated by **Figure** **1**a. Four different Ni—Co—S nanostructures (i.e., CoNi_2_S_4_, NiCo_2_S_4_, Ni—S, and Co—S) were prepared by one‐step hydrothermal process with different precursor ratios. The materials were freeze‐dried before use. Graphene oxide (GO) was prepared by a modified Hummer's method^12^ from graphite powders. Various hybrid materials were produced from mixtures of specific ratios of GO water solutions, thiourea (S and N source), and Ni—Co—S nanostructures in an autoclave at 180 °C for 12 h followed by a 3 d freeze‐drying process. Nitrogen‐doped graphene provides enhanced electron conductivity and ion electroactivity to materials. This is ascribed to the lone electron pairs from the nitrogen atoms forming delocalized conjugated systems with the sp^2^‐hybridized carbon frameworks.^35^ Sulfur doping was suggested to expand the highly efficient space utilization of carbon materials and improve the energy storage performances.^36^ The sulfur atoms are covalently incorporated into graphene and facilitated the bridging of metal sulfides materials to the graphene frameworks, thus enabling improved robustness of the composite materials.^37^ In addition, thiourea can not only support N and S co‐doping but also reduce the graphene oxide under the hydrothermal process thus further improving the electron conductivity. The digital camera pictures of the typical samples are shown in Figure 1b. Moreover, the as‐synthesized samples are superhydrophilic. The measured contact angle of a water droplet on the hybrid aerogel is 0° showing that it is fully wetted which is a highly beneficial characteristic for electrochemical reactions in aqueous electrolytes.

**Figure 2:**
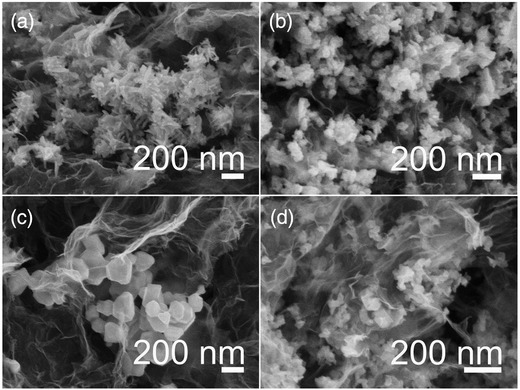
SEM pictures of a) CoNi_2_S_4_/SNGA, b) NiCo_2_S_4_/SNGA, c) Ni—S/SNGA, and d) Co—S/SNGA, respectively.

The morphologies of these materials were investigated by scanning electron microscopy (SEM). From the low‐magnification SEM images (Figure S1, Supporting Information), it can be observed that the Ni—Co—S nanoparticles were distributed uniformly on the surface of the porous graphene nanosheets. Porous channels can be observed for all four types of Ni—Co—S/SNGA. From the high‐magnification images (**Figure** **2**), the Ni—Co—S nanoparticles size could be determined. These particles were several tens of nanometers to hundreds of nanometers in size with a rounded particle shape or a nanorod shape within larger nanocluster structures. The small primary particle size together with the large surface contact area with the electrolyte is expected to accelerate the rate of redox reaction or catalytic actions.

**Figure 3:**
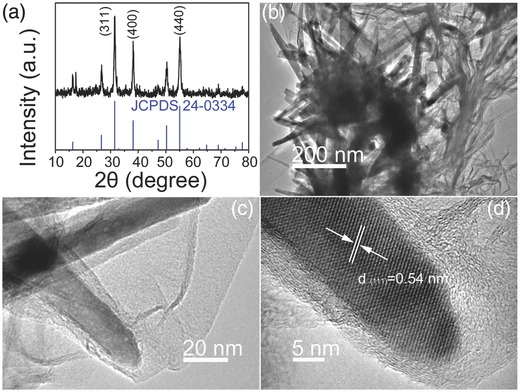
a) XRD patterns of CoNi_2_S_4_/SNGA and CoNi_2_S_4_ reference pattern; b,c) low and high‐magnification TEM images; and d) HRTEM of CoNi_2_S_4_/SNGA.

The crystallographic phases of the four types of Ni—Co—S nanostructures/SNGA were further characterized by powder X‐ray diffraction (XRD). **Figure** **3**a shows the XRD pattern of the CoNi_2_S_4_/SNGA samples, which correspond to the standard CoNi_2_S_4_ Fd‐3m crystal structures (JCPDS No. 24‐0334). XRD patterns of NiCo_2_S_4_/SNGA, Ni—S/SNGA, and Co—S/SNGA samples are shown in Figure S2b–d (Supporting Information), the peaks of which can be indexed to the standard diffraction patterns. The weak peaks at ≈24° in 2*θ* can be assigned as the (002) plane of SNGA. Figure 3b shows that the hydrothermally obtained CoNi_2_S_4_ nanostructures maintained a uniform urchin‐like morphology with nanorods self‐assembled radially and grown from the center, forming CoNi_2_S_4_ nanoclusters and capped by the graphene aerogel (GA), illustrated in Figure 2b. The individual nanorods have the diameter of ≈15 nm (Figure 3c). High‐resolution transmission electron microscopy (HRTEM) image of CoNi_2_S_4_/SNGA in Figure 3d showed clear lattice fringes with *d*‐spacing of 0.54 nm which can be indexed to the (111) plane of the Fd‐3m crystal structures of CoNi_2_S_4_, in accordance with the XRD results.

**Figure 4:**
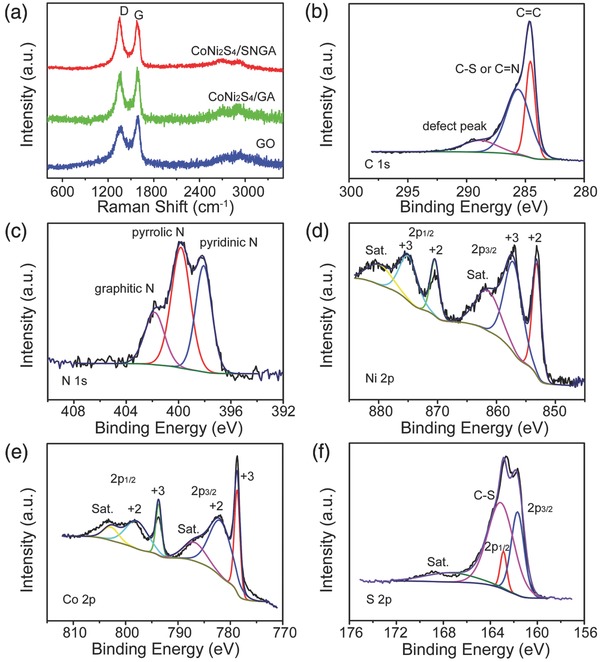
a) Raman spectra of as‐synthesized GO, CoNi_2_S_4_/GA, and CoNi_2_S_4_/SNGA, respectively; XPS spectra of b) C 1s, c) N 1s, d) Ni 2p, e) Co 2p, and f) S 2p spectra of CoNi_2_S_4_/SNGA sample, respectively.

Raman spectroscopy was performed to confirm the graphitization and composition of the GO, CoNi_2_S_4_/GA, CoNi_2_S_4_/S, N co‐doped graphene hybrid materials (CoNi_2_S_4_/SnrGO, Figure S3, Supporting Information) and CoNi_2_S_4_/SNGA samples, respectively. The two sharp peaks at 1353.2 and 1591.3 cm^−1^ correspond to the D and G peaks of GO and SNGA from the nanocomposites. The *I*(D)/*I*(G) value of the GO aerogel varied with the composition of the CoNi_2_S_4_ nanostructures and S, N co‐doping indicated that the degree of graphitization decreased with doping although the GO aerogel reduced to graphene.^10^ Among four different composition materials, CoNi_2_S_4_/SNGA possessed the highest *I*(D)/*I*(G) value (Table S1, Supporting Information), which proved that this sample has the highest reduction degree of GO which will produce more defects structures among graphene sheets than the other samples.^38^ The nature of the surface functionalities, along with the distributions of their valance states were analyzed by scanning electron microscopy combining with the energy‐dispersive X‐ray spectroscopy (SEM‐EDS mapping; Figure S4, Supporting Information) and X‐ray photoelectron spectroscopy (XPS). The sulfur was densely distributed within the structures, which demonstrated the S‐rich nature of the as‐synthesized samples. **Figure** **4**b shows the fitted C 1s peak of the XPS spectrum. The sharp peak at 284.4 eV was indexed to the sp^2^ C=C graphite bond. The peak at 285.6 eV was attributed to C—S and C=N bonding, indicating S and N co‐doped graphene and consistent with the Fourier transform infrared spectroscopy (FT‐IR) spectrum (Figure S5, Supporting Information). The weaker 288.3 eV peak is due to defects and functional groups including C—N, C—O, and O—C—O,^39^ the defect structures among graphene sheets can be directly related to the Raman results. The high‐resolution N 1s spectrum is shown in Figure 4c, and can be deconvoluted into three separated peaks: pyridinic N (398.1 eV), pyrrolic N (399.9 eV), and graphitic N (401.7 eV), respectively.^40^ The spectra of Ni 2p and Co 2p are shown in Figure 4d,e, and both of the Ni and Co elements can be fitted with two spin–orbit doublets and two shake‐up satellites. The doublets contain the low energy bands (Ni 2p3/2 and Co 2p3/2) and the high energy bands (Ni 2p1/2 and Co 2p1/2), respectively. This represented both the divalent and trivalent states of Ni and Co in the samples.^16^, ^19^ The S 2p spectrum (Figure 4f) can be divided into two main peaks located at ≈162.0 and 163.2 eV and one shake‐up satellite at ≈169.0 eV. The component at 163.2 eV corresponded to metal–sulfur bonds, and the peak at 162.0 eV can be attributed to the sulfur ions at low coordination numbers on the surface.^41^, ^42^ From XPS results of SNGA, the atom ratio of C 1s, S 2p, and N 1s are 88.76%, 4.76%, and 6.48%, respectively.

**Figure 5:**
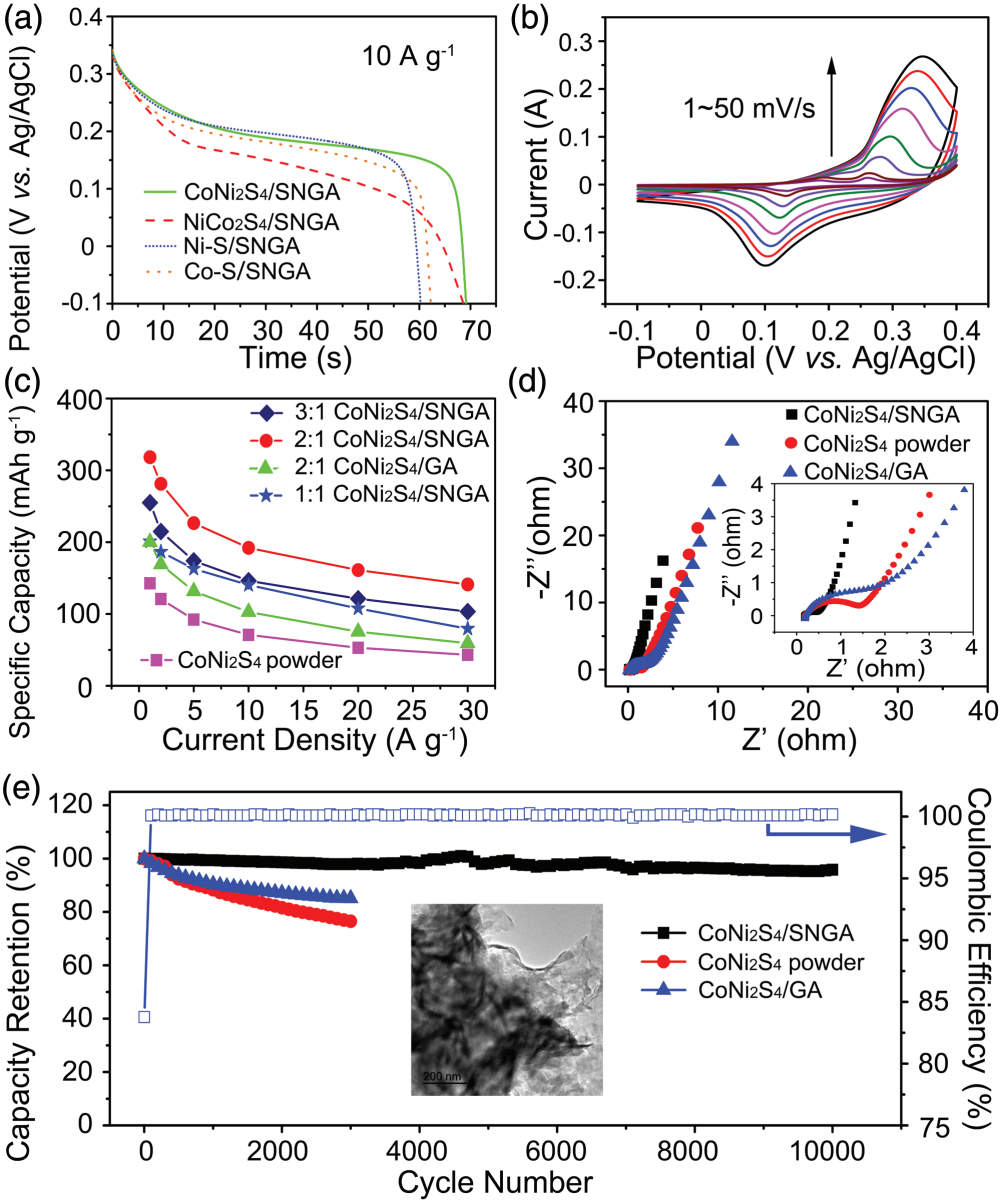
a) Discharge curves of CoNi_2_S_4_/SNGA, NiCo_2_S_4_/SNGA, Ni—S/SNGA, and Co—S/SNGA at the current density of 10 A g^−1^; b) cyclic voltammetry curves of CoNi_2_S_4_/SNGA at the scan rate of 1–50 mV s^−1^; c) the comparison of specific discharge capacity for different ratios of CoNi_2_S_4_/SNGA, CoNi_2_S_4_/GA, and CoNi_2_S_4_ powder samples as a function of current densities; d) Nyquist curves of CoNi_2_S_4_/SNGA, CoNi_2_S_4_/GA, and CoNi_2_S_4_ electrodes, inset showing high‐frequency parts of the EIS spectra for these samples; e) cycling performance of the CoNi_2_S_4_/SNGA for 10 000 cycles and CoNi_2_S_4_/GA and CoNi_2_S_4_ electrodes for 3000 cycles, respectively, inset showing the TEM image of the CoNi_2_S_4_/SNGA after long‐term cycling.

To explore the electrochemical performance of the as‐synthesized electrodes, we carried out cyclic voltammetry (CV) and galvanostatic charge–discharge (GCD) measurements on the as‐synthesized materials by pressing them directly into Ni foams as the working electrode without adding any conductive agents and binders in a three‐electrode test system with 6 m KOH as the electrolyte. We investigated the optimized performance of the samples by changing the ratio of nanoparticles to SNGA. From **Figure** **5**a, the specific capacity values of CoNi_2_S_4_/SNGA, NiCo_2_S_4_/SNGA, Ni—S/SNGA, and Co—S/SNGA were 192.1, 190.9, 167.5, and 184.0 mAh g^−1^, respectively, at a current density of 10 A g^−1^. The rate capabilities of those samples remained ≈44% of the original values, when the current density was increased by 30 times, as shown in Figure S7c (Supporting Information). The excellent specific capacity values and rate capabilities demonstrated the applicability of this rational design whereby different Ni—Co—S nanoparticles were investigated. The best performing material among the four tested as electrodes is the CoNi_2_S_4_/SNGA and the similar value can be seen from NiCo_2_S_4_/SNGA. This can be explained as the richer redox centers and the synergistic effect of both nickel and cobalt ions in the sulfides (redox reactions (1)–(3)). Figure 5b shows the CV curves of CoNi_2_S_4_/SNGA electrodes within the voltage range −0.1 to 0.4 V (vs Ag/AgCl) at scan rates ranging from 1 to 50 mV s^−1^.

As clarified by Dunn and co‐workers,^43^ if a redox reaction is limited by semi‐infinite diffusion like the battery type, the peak current *I* varies as *V* ^1/2^; for a capacitive process, it varies as *V*. This relation is expressed as *I* = *aV^b^*, with the value of *b* providing insight regarding the charge storage mechanism. In our system, the pairs of well‐defined redox peaks can be detected in all CV curves. The fitted value of *b* = 0.65 for the anodic peak current and *b* = 0.58 for cathodic peak current, the peak current *I* closely varies as *V* ^1/2^ (sweep rate) (detailed plot in Figure S8, Supporting Information), indicating these redox reactions are semi‐infinite diffusion. From the curves in Figure 5b, it can be observed that the anodic peaks shift toward positive potentials while the cathodic peaks shift toward negative potentials, respectively. The reaction is limited by the charge transfer kinetics. Increasing the scan rate, the redox peaks become obvious and only one pair of redox peaks can be observed in the curves (scan rate of 40 and 50 mV s^−1^), which can be explained as the polarization of the electrode at a high scan rate. These distinct peaks can be attributed to the reversible Faradaic redox processes of Ni^2+^/Ni^3+^ and Co^2+^/Co^3+^/Co^4+^ redox couples based on the following reactions (1–3)^28^

(1) $$\mathrm{NiS}+{\mathrm{OH}}^{-}\;\;\underset{}{⇄}\;\;\mathrm{NiSOH}+e^{-}$$

(2) $$\mathrm{CoS}+{\mathrm{OH}}^{-}\;\;\underset{}{⇄}\;\;\mathrm{CoSOH}+e^{-}$$

(3) $$\mathrm{CoSOH}+{\mathrm{OH}}^{-}\;\;\underset{}{⇄}\;\;\mathrm{CoSO}+H_{2}O+e^{-}$$

Because of these types of reversible redox reactions, we defined these materials in this reaction system for energy conversion and storage as rechargeable alkaline battery electrodes. Ex situ XRD measurements (Figure S9, Supporting Information) of CoNi_2_S_4_/SNGA samples during different electrochemical steps were carried out. The change in reflections implied the phase changing during this process and confirmed that redox reactions are occurring in bulk, further demonstrating the alkaline battery reactions.

GCD performances for the various synthesized Ni—Co—S/SNGA samples were conducted in the voltage ranges of −0.1 to 0.35 V (vs Ag/AgCl). The intrinsic properties of the CV curves (Figure 5b and Figure S7a, Supporting Information) and voltage plateaus observed during discharge curves (Figure 5a and Figure S6, Supporting Information) demonstrated obvious kinetic information so that these materials can be defined as rechargeable alkaline batteries electrodes, contrary to the previously reported materials which were classified as pseudocapacitor electrodes.^6^, ^19^, ^26^, ^28^, ^29^, ^42^, ^44^ The narrower voltage range of GCD compared with the CV curves is due to the fact that the electrode is not fully charged at low current densities (e.g., 1 A g^−1^). Therefore, the specific capacity values of the materials presented here are slightly lower compared to those recorded at a larger voltage range (see Figure S10, Supporting Information, and the calculation of specific capacity for CoNi_2_S_4_/SNGA at 20 A g^−1^ under different voltage ranges). When using different CoNi_2_S_4_ nanoparticles and SNGA ratios, i.e., 0.14 g of nanoparticles in 70 mg of GO precursors (2:1 CoNi_2_S_4_/SNGA), a high specific capacity and rate capacity were achieved compared with other ratios tested. The specific capacity was 318.3, 281.3, 226.5, 192.1, 161.1, and 141.2 mAh g^−1^ at current densities of 1, 2, 5, 10, 20, and 30 A g^−1^, respectively. The rate capability was ≈44.3% when the current density was increased 30 times. The best performance of the 2:1 CoNi_2_S_4_/SNGA is as the result of an optimum ratio of active materials and porosity. Therefore, all of the other experiments were conducted using this ratio unless stated otherwise. Compared with the CoNi_2_S_4_ nanoclusters and the same ratio CoNi_2_S_4_/GA, 1:1 and 3:1 CoNi_2_S_4_/SNGA samples exhibited higher specific capacity at all current densities.

The electronic conductivities of CoNi_2_S_4_/SNGA, CoNi_2_S_4_/GA, and CoNi_2_S_4_ nanoclusters are significant parameters and can be used to explain the differences in performances between the various materials. Electrochemical impedance spectra (EIS) were employed as depicted in Figure 5d. The Nyquist plots had a semicircle in the high‐to‐medium frequency region (inset in Figure 5d) and a slope in the low frequency region. The semicircle is attributed to charge transfer processes at the electrode/electrolyte interface, while the plot corresponds to electrolyte diffusion processes into the bulk of the electrode, i.e., Warburg diffusion.^44^, ^45^ In the case of CoNi_2_S_4_/SNGA material, the slope was more abrupt indicating that the Warburg resistance (*Z*_w_, diffusive impedance of the OH^−^ ion) appears not to be the determining factor. This material can therefore store charge more efficiently when used as an electrode.

Figure S11 (Supporting Information) shows the modified equivalent circuit model of our system, the equivalent series resistance (*R*_s_) values, including inherent resistances of the active materials, bulk resistance of electrolyte, and contact resistance of the interface between electrolyte and electrodes. The *R*_s_ values were 0.155, 0.192, and 0.185 Ω for CoNi_2_S_4_/SNGA, CoNi_2_S_4_/GA, and CoNi_2_S_4_ nanoclusters, respectively (well‐fitted EIS spectra were shown in Figure S12, Supporting Information). The relatively low values are similar for all three samples, which suggest the spinel structures of CoNi_2_S_4_ nanoparticles could possess metallic electronic conductivity.^31^ GA with the lower reduction degree of GO could result in a decline of the conductivity of the whole structure. The charge‐transfer resistance (*R*_ct_) values, calculated from the semi‐circle in the high‐frequency region, reflect the diffusion of electrons, and are 0.43, 2.02, and 1.37 Ω for CoNi_2_S_4_/SNGA, CoNi_2_S_4_/GA, and CoNi_2_S_4_ nanoclusters, respectively. The lower capacity value of CoNi_2_S_4_ powder samples can be explained by the electrode manufacture procedure (without adding binder), which can lead to leakage and aggregation of the materials that were immersed into the electrolyte especially under the working state. The CoNi_2_S_4_/SNGA system possessed the smallest resistance among its other counterparts; its electronic conductivity is the determining factor for the high‐performance rechargeable alkaline battery electrode.

Lou and co‐workers reported that the surface of metal sulfides can be electrochemically transformed to metal hydroxides upon repeated cycling processes.^28^ Indeed, the biggest challenge related to metal sulfides materials when used in energy storage is their unsatisfactory cyclability, due to the oxidation of sulfide materials and the decline of conductivity especially after long‐term cycling. Here, we have conducted long‐term cyclic GCD tests of 10 000 cycles for the as‐synthesized CoNi_2_S_4_/SNGA at a current density of 10 A g^−1^. The specific capacity of the 10 000th cycle was ≈95.8% of its first cycle value. The Coulombic efficiency of the sample stayed at ≈100% except for the first few cycles, meaning an excellent reversible redox process. CoNi_2_S_4_/SNGA samples in our work for the three‐electrode test proved to have superior specific capacity and cycling performances among the most of the reported for nickel/cobalt sulfide materials (see Table S2, Supporting Information, for comparison). For comparison, the specific capacity value of the 3000th cycle of CoNi_2_S_4_/GA, CoNi_2_S_4_/NGA, and CoNi_2_S_4_/SGA was ≈85.1%, 77%, and 93% of its initial value under the same test conditions; moreover, the S element signal in XPS for CoNi_2_S_4_/GA and CoNi_2_S_4_/NGA could hardly be detected (Figures S13 and S14d, Supporting Information). The specific capacity value of the 3000th cycle for CoNi_2_S_4_ powdery sample was ≈76.5% of its initial values after cycling. The TEM mapping images (Figure S15, Supporting Information) and XPS data (Figure S16, Supporting Information) after cycling confirmed the composition and structure stability of the CoNi_2_S_4_/SNGA samples. Sulfur and nitrogen can also be detected from the elemental mapping images from the TEM and metal–sulfide binding can be inferred from the XPS data, which indicates that the CoNi_2_S_4_/SNGA structure is very stable and can slow down the electrochemical transformation of the metal sulfides to metal hydroxides. The morphology of CoNi_2_S_4_ nanorods became rough (TEM image in inset of Figure 5e) due to the long time electrochemical reactions. However, the CoNi_2_S_4_ nanostructures were still capped in the initial SNGA structures. The impedance spectra (Figure S17, Supporting Information) after the cycling showed the *R*_s_ and *R*_ct_ are 0.294 and 1.85 Ω, respectively, which increased fractionally compared with their initial values, and have comparable or slightly smaller values compared with the reported similar binder‐free metal sulfide materials before cycling.^19^, ^44^, ^46^ Similar cycling performances can also be observed for NiCo_2_S_4_/SNGA, Ni—S/SNGA, and Co—S/SNGA samples. The 3000th cycles of all these four samples possessed capacity retention higher than ≈89% (Figure S7d, Supporting Information). Moreover, the specific capability and low resistance (Figure S7b,c, Supporting Information) indicate these Ni—Co—S/SNGA possess high performance. By comparing the electrochemical performances of these samples, the functions of S and N doping can be clarified. Nitrogen doping could be used to improve the electron conductivity and ion electroactivity of the graphene aerogel and sulfur doping can help stabilize the metal sulfides. These evidences proved the general usability of this structure to improve the performances of the Ni—Co—S materials used for rechargeable alkaline batteries electrodes, especially the cycling performances.

**Figure 6:**
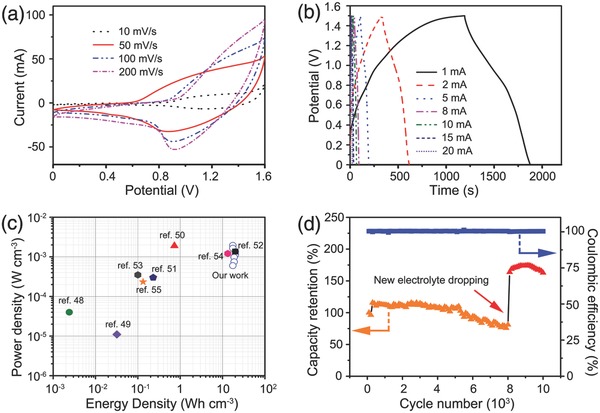
a) CV curves for the hybrid battery (SNGA//CoNi_2_S_4_/SNGA) at the scan rate of 10, 50, 100, and 200 mV s^−1^, respectively; b) GCD curves at the current of 1, 2, 5, 8, 10, and 20 mA, respectively; c) Ragone plot of the hybrid battery and compared with some devices values in literature; d) cycling performance of the devices at a current of 20 mA.

In order to evaluate the CoNi_2_S_4_/SNGA electrode for practical applications, a solid‐state hybrid battery was fabricated by using the CoNi_2_S_4_/SNGA pressed into nickel foam as the positive electrode, the SNGA pressed into nickel foam as the negative electrode, 2 m KOH‐PVA solution as the gel electrolyte, and commercial glassy fiber paper as the separator. Solid‐state hybrid battery has similar structures as reported previously to so‐called asymmetric supercapacitors. The electrochemical reactions at the anode were as stated by reactions (2)–(4). The negative electrode is a graphene aerogel based electrical double layer capacitor (EDLC); it has the mechanism of electrostatic adsorption for energy storage at the interface of the electrodes. The device is based on the electrodes of rechargeable alkaline battery and EDLC; we defined it as a hybrid battery, as they possess obvious redox peaks with semi‐infinite diffusion reactions. **Figure** **6**a displays the CV curves of the solid‐state hybrid battery (SNGA//CoNi_2_S_4_/SNGA) at various scan rates (10–200 mV s^−1^) in the voltage range of 0–1.6 V. Clearly, the CV curves showed hybrid capacity of both an electric double‐layer capacitor and a redox reaction of a battery. The volumetric specific capacity of the devices was calculated from the GCD curves (Figure 6b). The fabricated SNGA//CoNi_2_S_4_/SNGA device processed the volumetric specific capacity of 2.37, 1.99, 1.49, 1.24, 1.14, 0.92, and 0.76 mAh cm^−3^ at current densities of 1, 2, 5, 8, 10, and 20 mA cm^−2^, respectively. From the discharge curve, it possess unconspicuous voltage plateaus indicating the pseudocapacitor‐like hybrid feature. Energy density and power density are two key metrics for evaluating the performances of the energy storage device. Figure 6c shows the plots of power density versus energy density (Ragone plot) of the solid‐state hybrid battery on the basis of the total volume of the device. The volumetric energy density and power density of the hybrid battery were calculated according to the well‐defined equations in Equations (S4)–(S10) (Supporting Information). The device exhibited a high volumetric power density (maximum output value) of 17.5 W cm^−3^ while retaining its high energy density of 1.95 mWh cm^−3^ at a current of 1 mA. This value remained almost constant with increasing current, which is comparable or better than most of the reported symmetric or asymmetric devices (SDs or ADs)^47^ based on the volumetric density, such as ZnO@C@MnO_2_‐SDs,^48^ TiO_2_@C‐SDs,^49^ WO_3−_ *_x_*/MoO_3−_ *_x_*//PANI/carbon fabric‐Ads,^50^ H‐TiO_2_@MnO_2_//H‐TiO_2_@C‐ADs,^51^ laser‐scribed graphene SDs,^52^ MnO_2_//Fe_2_O_3_ ADs,^53^ Co_9_S_8_//Co_3_O_4_@RuO_2_‐ADs,^54^ and ZnO@MnO_2_//RGO‐ADs.^55^ The energy density and power density based on the total mass of the hybrid battery are shown in Figure S19 (Supporting Information). The high power density value was retained showing a superior behavior to some of the literature reported hybrid devices based only on the caculation of the active materials.^56^, ^57^ The cycling performances for the hybrid battery are shown in Figure 6d at a current of 20 mA. It manifested a high cycling stability and overall capacity decrease of only ≈0.002% cycle^−1^ within 8000 cycles. The Coulumbic efficiency of the hybrid battery stayed at ≈100%. The cycling performances are better than or comparable to the similar PVA gel electrolyte based solid‐state devices (Table S3, Supporting Information, for comparison). Moreover, with the new gel electrolyte dropping on the solid‐state hybrid battery, the capacity increased dramatically with several hundreds of cycles increasing together with the internal resistance change (Figure S20, Supporting Information), indicating the main reason for the decline in performance is further water evaporation in the gel electrolyte thus causing the internal resistor to increase and limiting the contacting reaction sites with the active materials.

**Figure 7:**
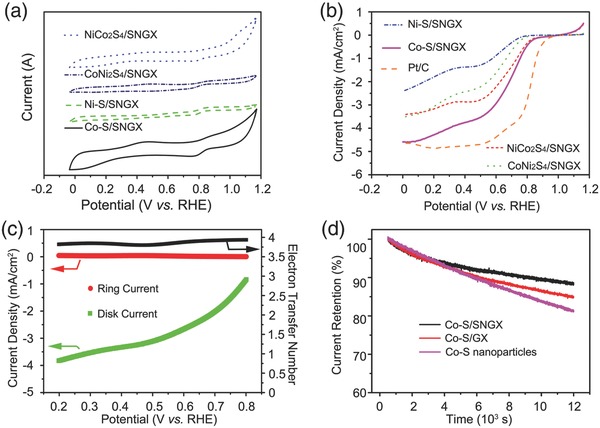
a) CV curves of Ni—Co—S/SNGA in O_2_‐saturated 0.1 m KOH at room temperature; b) rotating‐disk electrode (RDE) measurement of Ni—Co—S in O_2_‐saturated 0.1 m KOH at 1600 rpm with a sweep rate of 10 mV s^−1^; c) RRDE measurement of Co—S/SNGA. Two curves representing the current density on the disk (*i*_disk_, green curve) and ring (*i*_ring_, red curve) electrodes, respectively, and the electron transfer number (black curve); d) 12 000 s current–time chronoamperometric responses of Co—S/SNGA, Co—S/GA, and Co—S nanostructures at a rotation rate of 1600 rpm in O_2_‐saturated 0.1 m KOH.

Ni—Co—S ternary systems can also be used as electrocatalyts as they have noble‐metal‐like catalytic properties, while their stability should be influenced when used in an alkaline electrolyte especially in O_2_ saturated KOH. Thus, we have also explored the use of Ni—Co—S/SNGA materials as ORR catalysts. **Figure** **7**a shows the CV of Ni—Co—S/SNGA in O_2_‐saturated 0.1 m KOH at room temperature. The peak potential of Co—S/SNGA nanostructures (0.75 V vs RHE) is more positive than other Ni—Co—S/SNGA nanostructures, with similar values of NiCo_2_S_4_/SNGA (0.746 V vs RHE). The ORR performances of the four materials evaluated based on Ni—Co—S/SNGA nanostructures were further compared by rotating‐disk electrode (RDE) measurement in O_2_‐saturated 0.1 m KOH at 1600 rpm with a sweep rate of 10 mV s^−1^, as shown in Figure 7b and Figure S21a (Supporting Information). The Co—S/SNGA nanostructure showed the best performance compared with other Ni—Co—S/SNGA nanostructures, Co—S nanoparticles, and Co—S/GA, with the onset potential of 1.0 V versus RHE and limiting current density of 4.6 mA cm^−2^. As reported, with the increase of Ni^3+^ parts in Ni—Co—S, the ORR catalysts show poorer performance.^58^ The electron transfer number of Co—S/SNGA ranged from 3.8 to 3.95 during the voltage range of 0.2–0.8 versus RHE, revealing the four‐electron pathway reaction. Similar results can be seen from NiCo_2_S_4_/SNGA from Figure S21b (Supporting Information), while the electron transfer number remained stable at 3.8–3.95, indicating a four‐electron pathway of this sample. The CoNi_2_S_4_/SNGA material also showed a four‐electron type reaction while the Ni—S/SNGA showed a mixed two‐ and four‐electron hybrid pathway. With an increase of Ni ion in the hybrid structures, the ORR performance decreased. Moreover, from the comparison experiments (Figure S22, Supporting Information), the ORR performances of NiCo_2_S_4_/SNrGO are similar to that of NiCo_2_S_4_/SNGA, which indicates that the aerogel structures have almost no influence on the interaction of each component.

Stability is another important index to evaluate the performance of ORR catalysts. As expected, the SNGA wrapped Co—S nanostructures showed better performance, and maintained ≈88.6% of the initial value after 12 000 s of current–time chronoamperometric responses at a rotation rate of 1600 rpm in O_2_‐saturated 0.1 m KOH, higher than Co—S nanoparticles and Co—S/GA. The usability of these structures can also be evidenced by NiCo_2_S_4_/SNGA, with the ≈91.5% retention (Figure S23, Supporting Information) after 12 000 s in the current–time chronoamperometric responses.

## 3 Conclusion

In summary, we have developed Ni—Co—S/SNGA materials as a viable alternative to the existing materials to improve the electrochemical performances especially the cycling performances of Ni—Co—S ternary metal sulfide materials for both rechargeable alkaline battery electrodes and ORR catalysts. The CoNi_2_S_4_/SNGA samples showed the best performance, which delivered a high specific discharge capacity of 318.3 mAh g^−1^ at 1 A g^−1^. The capacity can retain 44.3% of the initial value when the current density increased 30 times. The capacity retention of ≈95.8% of the initial specific capacity after a long GCD cycle of 10 000. The high‐rate hybrid battery based on CoNi_2_S_4_/SNGA as the positive electrode and SNGA as the negative electrode delivered a high volumetric power density of 17.5 W cm^−3^ while it retained its high volumetric enegy density of 1.95 mWh cm^−3^ at the current of 1 mA. The hybrid battery showed an excellent cycling performance and the capacity decrease was only ≈0.002% cycle^−1^ within 8000 cycles. When used as electrocatalysts in ORR, it was observed that an increase of Ni ion in the hybrid structures decreased the performance. Remarkably, SNGA structures capped with metal sulfide represent enhanced stability for ORR. Moreover, we confirmed the commonly regarded supercapacitor electrodes based on Ni—Co—S ternary materials as electrode in rechargeable alkaline batteries from a kinetic view. We have also proposed accurate caculation formulas to evaluate the performance of the hybrid battery devices, which could serve as a standardization for performance comparisons in future work for hybrid batteries.

The excellent performance of our materials is due to an increased stability and resistance against oxidation rendering them potential candidates for other applications such as metal‐ion and Li—S batteries and other electrocatalysis like oxygen evolution reaction catalysis.

## Experimental Section

Detailed experimental sections can be found in the Supporting Information.

1. The synthesis of four kinds of nickel cobalt sulfide nanostructures; graphene oxide; nickel cobalt sulfide/S, N co‐doped reduced graphene oxide hybrid materials; S, N doped graphene‐based nickel cobalt sulfide aerogel; and S, N co‐doped graphene aerogel;
2. Materials characterization;
3. Electrochemical properties testing and devices fabrication.

## Supporting information

As a service to our authors and readers, this journal provides supporting information supplied by the authors. Such materials are peer reviewed and may be re‐organized for online delivery, but are not copy‐edited or typeset. Technical support issues arising from supporting information (other than missing files) should be addressed to the authors.

Supplementary
