# Supplementary material for: S, N‐Co‐Doped Graphene‐Nickel Cobalt Sulfide Aerogel: Improved Energy Storage and Electrocatalytic Performance
Source: Adv Sci (Weinh). 2016 Aug 17;4(1):1600214. doi: 10.1002/advs.201600214 (PMC5238742; doi:10.1002/advs.201600214)
Supplement: Supplementary file 1 — Supplementary [file ADVS-4-0-s001.pdf]

Copyright WILEY-VCH Verlag GmbH & Co. KGaA, 69469 Weinheim, Germany, 2016.

ADVANCED SCIENCE Open Access

Supporting Information

for *Adv. Sci.,* DOI: 10.1002/advs.201600214

S, N-Co-Doped Graphene-Nickel Cobalt Sulfide Aerogel: Improved Energy Storage and Electrocatalytic Performance

*Guanjie He, Mo Qiao, Wenyao Li, Yao Lu, Tingting Zhao, Rujia Zou, Bo Li, Jawwad A. Darr, Junqing Hu, Maria-Magdalena Titirici, and Ivan P. Parkin**

Supporting Information

S, N-co-doped Graphene-Nickel Cobalt Sulfide Aerogel: Improved Energy Storage and Electrocatalytic Performance

*Guanjie He[a], Mo Qiao[b], Wenyao Li[c], Yao Lu[a], Tingting Zhao[a], Rujia Zou[c], Bo Li[d], Jawwad A. Darr[a], Junqing Hu[c], Maria-Magdalena Titirici[b] and Ivan P. Parkin[a]**

Guanjie He, Yao Lu, Tingting Zhao, Prof. Jawwad A. Darr, Prof. Ivan P. Parkin
[a]Christopher Ingold Laboratory, Department of Chemistry, University College London, 20 Gordon Street, London WC1H 0AJ, U.K.,
E-mail:i.p.parkin@ucl.ac.uk
Mo Qiao, Prof. Maria-Magdalena Titirici
[b]School of Engineering and Materials Science/Materials Research Institute, Queen Mary University of London, Mile End Road, E14NS, London, UK.
Dr. Wenyao Li, Prof. Rujia Zou, Dr. Bo Li, Prof. Junqing Hu
[c]State Key Laboratory for Modification of Chemical Fibers and Polymer Materials, College of Materials Science and Engineering, Donghua University, Shanghai 201620, China.

Experimental parts:

Graphite powders were purchased from Hopkin & Williams Company (U.K.). Ni foams were from the Suzhou JSD foam metal Co. Ltd. (China). The glassy fiber papers were purchased from Fisher Scientific (U.K.). PVA was bought from Tokyo Chemical Industry Co., Ltd. All the other chemicals were purchased from Sigma-Aldrich (U.K.) or VWR International (U.K.) and used as received without further purification.

1. Synthesis of four different ratios of nickel cobalt sulfide nanostructures

Four 50 mL mixtures with different molar ratios of $Ni(NO_3)_2$•$6H_2O$ and $Co(NO_3)_2$•$6H_2O$ (1st beaker: $Ni(NO_3)_2$•$6H_2O$ (1 mmol) and $Co(NO_3)_2$•$6H_2O$ (2 mmol); 2nd beaker: $Ni(NO_3)_2$•$6H_2O$ (2 mmol) and of $Co(NO_3)_2$•$6H_2O$ (1 mmol); 3rd beaker: $Ni(NO_3)_2$•$6H_2O$ (3 mmol); 4th beaker: $Co(NO_3)_2$•$6H_2O$ (3 mmol) were prepared. The nickel and cobalt precursors were dissolved in deionized (DI) water (15 mL) and ethanol (20 mL) with magnetic stirring for 1 hour. Then, 1 g of thiourea was dissolved in the above solutions sequentially with another 1 hour stirring. Finally, the pH of each solution was adjusted to 8.0 using ammonium hydroxide solution (ACS reagent, 28 - 30% NH_3 basis). The solutions were transferred into 50 mL autoclaves and kept in the electric oven at 180 ^oC for 12 h.

2. Synthesis of graphene oxide

Graphene oxide (GO) was synthesized from graphite powder by the improved Hummer's methods as reported with a few adjustment.[1] Firstly, graphite powder (3 g) was added into the 98 % sulfuric acid (69 mL) in an 800 mL beaker with 145 r/min magnetic stirring for 1 h. After, potassium permanganate (9 g) was added into the above suspension during ~ 45 min. The beaker was then kept in a 35 ^oC oil bath for 2 h without stirring. The temperature of the reaction system was adjusted from 35 ^oC to 98 ^oC and DI water (138 mL) was added into the reaction system slowly and kept at 98 ^oC for 15 min during this process. Additional water (420 mL) together with preheated H_2O_2 (30%) (7.5 mL) was added while the color of the solution changed from dark brown to light yellow. The solution was kept at room temperature with magnetic stirring for 24 h. Subsequently, the powder was filtered and washed with HCl solution (H_2O (750 mL) and concentrated HCl (7.5 mL) to remove metal ions). The filter cake was re-dispersed in the DI water and the aggregates were removed from the dispersion by 15 min of centrifugation at 3000 rpm. Finally, the dispersion was purified by dialysis for three days to remove any salt impurities.

3. Synthesis of S, N doped graphene-based nickel cobalt sulfide aerogel (Ni-Co-S/SNGA) and S, N co-doped graphene aerogel (SNGA).

Briefly, the GO dispersion solution was adjusted to ~ 2 mg/ml. Different ratios of nickel cobalt sulfide nanoparticles (2:1 ratio is 0.14 g from step 1) were added into four beakers containing GO solution (35mL, ~ 2 mg/ml) respectively with magnetic stirring for 1 h. Thiourea (0.5 g) was added to each beaker with another 1 h magnetic stirring. At last, the above solutions were transferred to four 50 ml autoclaves and kept in the preheated oven at 180 ^{o}C for 12 h. The as-synthesized hydrogels were placed into the beakers with DI water (50 mL; Changing water every half day) for three days to remove the impurities and freeze-dried at ~ -50°C for three days. SNGA were prepared by the same process without adding Ni-Co-S nanoparticles at the first step. For comparison, N-doped graphene based $CoNi_2S_4$ and S-doped graphene based $CoNi_2S_4$ were synthesized by using the same ratio with urea (0.4 g) and sodium sulphide (0.5 g) as the N and S sources, respectively.

4. Synthesis of nickel cobalt sulfide /S, N co-doped reduced graphene oxide hybrid materials

Firstly, certain amount of the (0.2 M $Co(NO_3)_2$, 0.525 mL and 0.1 M $Co(NO_3)_2$, 0.525 mL for $NiCo_2S_4$/SNrGO synthesis) aqueous solutions were added to 4 ml of GO water suspension (~ 2 mg/ml), followed by the addition of water (6 mL) and ethanol (6 mL), the PH of the solution was adjusted to ~ 8. The reaction was kept at 80 ^{o}C for 12 h with stirring. In the second step, thiourea (0.15 g) was added into reaction mixture with magnetic stirring for 1 h. Finally, the solution was transferred to a 25 ml autoclave for solvothermal treatment at 180°C for 12 h.

Characterization: The morphology and microstructure of samples were characterized by scanning electron microscopy (SEM, Hitachi S-4800; SEM, JSM-6700F equipped with an

energy-dispersive X-ray spectrometer) and TEM (JEOL, JEM-2100). The phase and chemical composites were recorded using a D4 ENDEAVOR X-ray diffractometer (XRD; Cu-Ka radiation), an X-ray photoelectron spectroscopy (XPS; Thermo scientific K-alpha photoelectron spectrometer), Raman Spectroscopy (Renishaw Raman microscope spectrometer with the laser wavelength of 514.5 nm) and Attenuated Total Reflectance Fourier transform infrared spectroscopy (ATRFTIR, BRUKER, platinum-ATR). *Ex-situ* XRD for the charge and discharge processes of the alkaline battery electrodes were recorded on a STOE SEIFERT diffractometer (Mo source radiation). The mass of the materials for electrodes and active materials used for ORR test was weighed accurately by an analytical balance (Ohaus; δ = 0.01 mg).

Electrochemical analysis:

Alkaline battery electrodes' performance testing. Electrochemical measurement of the as-synthesized electrodes were performed on a Gamry electrochemical workstation (Gamry Interface 1000) in a three-electrode cell with 6 M KOH as the electrolyte. A platinum foam (~ 2 cm^2) and an Ag/AgCl electrode were used as the counter and reference electrode, respectively. The active materials were pressed into Ni foams as binder-free and conductive reagent-free working electrodes. The mass of the active materials on nickel foam is ~ 2 mg (area of ~ 1 cm^2) for testing. The electrochemical impedance spectroscopy measurements were performed at open circuit potential with a sinusoidal signal over a frequency range from 100 kHz to 0.01 Hz at an amplitude of 10 mV. The specific capacity was calculated by the equation as follows:

$$C = \frac{I \times \Delta t}{m} \quad (1)$$

I is the applied galvanostatic current for the charge-discharge test (A). Δt is the discharge time (s), m is the mass of the active material (g). The values were calculated after 100 cycles of cyclic voltammetry (CV) at 50 mV s^{-1} for activation.

Hybrid battery devices fabrication and evaluation.

Electrolyte preparation: The electrolyte was prepared by mixing two kinds of solutions: a. 6 g of PVA was dissolved into 40 mL of DI water at the temperature of 90 ^{o}C, b. 20 mL of 6 M KOH water solution. The mixture was transferred to the room temperature with magnetic stirring for further use.

Devices fabrication: Hybrid batteries were fabricated by pressing the $CoNi_2S_4$/SNGA into the Ni foam (~ 1 cm^2) as the positive electrode and the SNGA into the Ni foam (~ 1 cm^2) as the negative electrode. The mass ratio of the positive to negative electrode was calculated according to the charge balance equation (Q_+=Q_-). Before fabrication, the electrodes were activated by CV tests at the scan rate of 50 mV s^{-1} in the 6 M KOH. The fabricated devices were left in the fume hood for 2 h to let the extra water evaporation and gel formation. Single device needs ~ 0.5 mL of PVA/KOH solutions. PVA was calculated as ~ 0.05 g of PVA in each device.

In order to satisfy the $Q_+ = Q_-$, the mass ratio (active materials) of two electrodes were decided by the following equation:

$$\frac{m_+}{m_-} = \frac{C_-}{C_+} \qquad (2)$$

C_+ and C_- (mAh g^{-1}) are the mass specific capacity of the $CoNi_2S_4$/SNGA and SNGA, respectively. The mass ratio was calculated as *ca.* 1:7.

The specific capacity of the device (C_{device}) was calculated from the galvanostatic charge-discharge (GCD) curve as:

$$C_{device,volume} = \frac{I \times \Delta t}{V} \quad (3)$$

Where $C_{device,volumn}$ is the volumetric specific capacity based on the volume calculation of the whole device (mAh cm^{-3}). I is the applied galvanostatic current for the charge-discharge test (A). Δt is the discharge time (s) for the device. V is the total volume for the device (cm^3).

The energy density (E_{volume} or E_{mass}) and the power density (P_{volume} or P_{mass}) based on the whole volume or mass of the device were calculated based on the following equation:

$$E = I \int_0^{t_{max}} U_{(t)} dt \quad (4)$$

$$P_{output,max} = \frac{U^2}{4R_s} \quad (5)$$

$$R_s = \frac{U_{drop}}{2I} \quad (6)$$

$$E_{volume} \ or \ E_{mass} = \frac{E}{V_{device}} \ or \ \frac{E}{m_{device}} \quad (7)$$

$$P_{volume} \ or \ P_{mass} = \frac{P_{output,max}}{V_{device}} \ or \ \frac{P_{output,max}}{m_{device}} \quad (8)$$

Where E is the total energy from the device, t_{max} is the total discharge time, U is the voltage range of GCD test for the device (excluding the voltage drop from the beginning of the discharge curve), R_s is the internal resistance of the device, U_{drop} is the voltage drop from the discharge curve and I is the current from the galvanostatic test. P_{output}, I_{output} and R_e are the power, current and resistant of the external circuit.

The equation (5) was from:

$$I_{output} = \frac{U}{R_e + R_s} \quad (9)$$

$$P_{output} = {I_{output}}^2 R_e = \frac{U^2}{R_e + R_s} \times \frac{R_e}{R_e + R_s} = \frac{U^2}{R_e + \frac{{R_s}^2}{R_e} + 2R_s} = \frac{U^2}{\frac{(R_e - R_s)^2}{R_e} + 4R_s} \quad (10)$$

When $R_e = R_s$, the P_{output} can show the maximum value.

1 cm^2 device including: nickel foam, active materials, separator and gel electrolyte, the total mass: 0.24222 g; the total volume: 0.08 cm^3, the thickness was evaluated by the vernier caliper (Resolution: 0.02 mm).

Oxygen reduction reaction measurement. In a typical ORR performance test, the electrodes were prepared by mixing 4 mg of catalysts, 964 µL of DI water and 36 µL of nafion solution (5 % w/w). The mixture was then sonicated for ~ 40 min in the ice bath to obtain a homogeneous ink. For preparing rotating disk electrode (RDE), 5 µL of the slurry was then deposited onto the glassy carbon disk (3 mm in diameter) of RDE and dried for ~ 30 mins under room temperature in air. 14 µL of the same ink was applied to prepare for the rotating ring-disk electrode (RRDE, 5 mm in diameter). All tests were performed on electrochemical workstation (Metrohm Autolab PGSTAT204) in a standard three-electrode cell. The Ag/AgCl electrode and the platinum wire were used as the reference and the counter electrode, respectively. CV was performed in oxygen-saturated 0.10 M KOH solution at a scan rate of 100 mV s^{-1}. Linear sweep voltammograms (LSV) were obtained by rotating the electrode at 400, 800, 1200, 1600, 2000 and 2400 rpm respectively at a scan rate of 10 mV s^{-1} in oxygen-saturated 0.10 M KOH solution.

The analysis of transferred electron number per O_2 in the ORR procedure was based on：

$$n = \frac{4I_d}{I_d + \frac{I_r}{N}} \quad (11)$$

Where I_d is the disk current, I_r is the ring current and N is current collection efficiency of the Pt ring. In our testing system, N was tested to be 0.25 from the reduction of $K_3Fe[CN]_6$. The current-time chronoamperometric responses was recorded after the first 500 s activation period for stabilizing the electrodes at the -0.45 V *vs.* Ag/AgCl at a rotation rate of 800 rpm in O_2-saturated 0.1 M KOH.

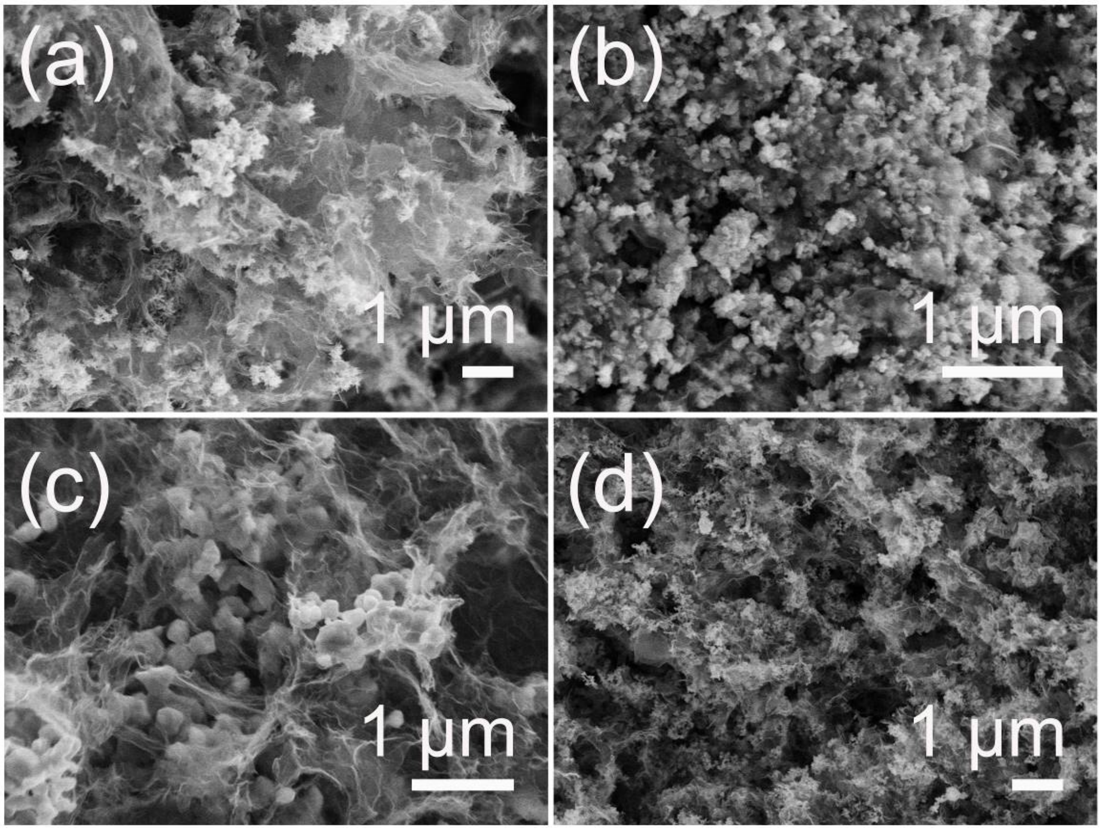

Figure. S1 (a-d) Low-magnification SEM pictures of $CoNi_2S_4$/SNGA, $NiCo_2S_4$/SNGA, Ni-S/SNGA, Co-S/SNGA, respectively.

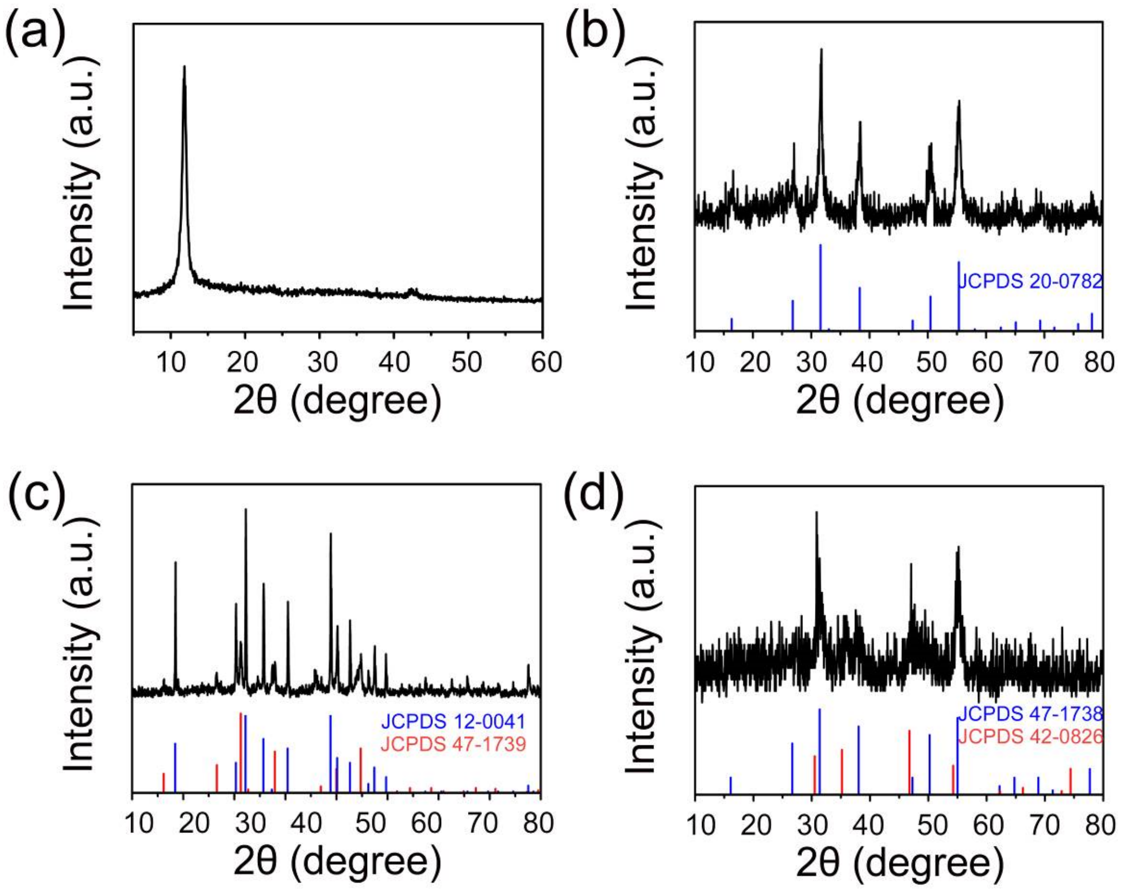

Figure. S2 XRD patterns of (a) GO; (b-d) as-synthesized Ni-Co-S/SNGA nanostructures: (b) $NiCo_2S_4$/SNGA, (c) Ni-S/SNGA and (d) Co-S/SNGA and their standard patterns, respectively.

The crystal phase of the GO and Ni-Co-S/SNGA nanostructures can be determined by XRD, The sharp peak at around $2\theta = 10.8^o$ corresponds to the (002) reflection of GO and disappeared in the patterns of reduced samples.[2] The $NiCo_2S_4$ nanoparticles in $NiCo_2S_4$/SNGA is the pure phase, corresponds to the Fd-3m crystal structures of standard $NiCo_2S_4$ (JCPDS no. 20-0782). By simply changing the ratio of Ni-Co-S precursors, we can obtain dual phases of Ni-S and Co-S nanoparticles, which can be indexed to the Ni_3S_4 (JCPDS no. 47-1739), NiS (JCPDS no. 12-0041) and Co_3S_4 (JCPDS no. 47-1738), $Co_{1-x}S$ (JCPDS no. 42-0826), respectively.

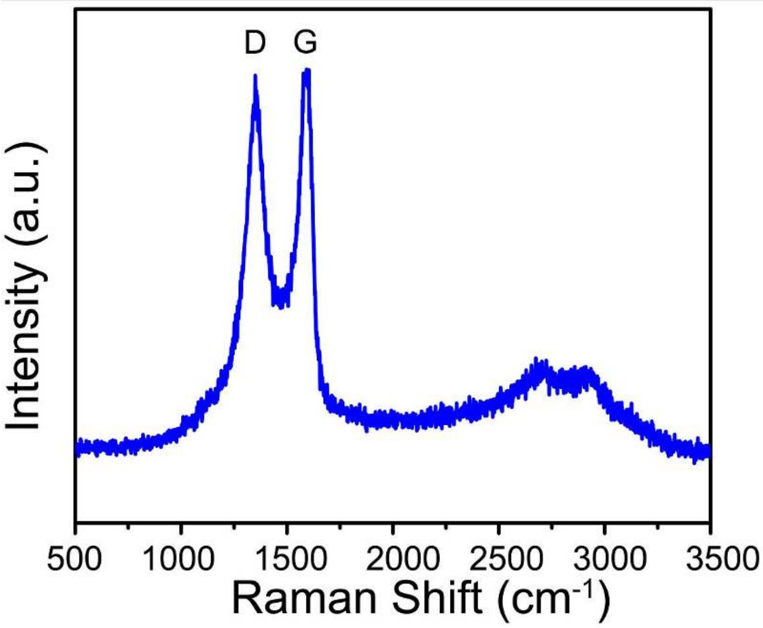

Figure. S3 Raman spectrum of the $CoNi_2S_4$/SNrGO

Table. S1 I(D)/I(G) values of the different samples.

Samples	I(D)/I(G) values
GO	0.80
$CoNi_2S_4$/SNrGO	0.91
$CoNi_2S_4$/GA	0.89
$CoNi_2S_4$/SNGA	1.05

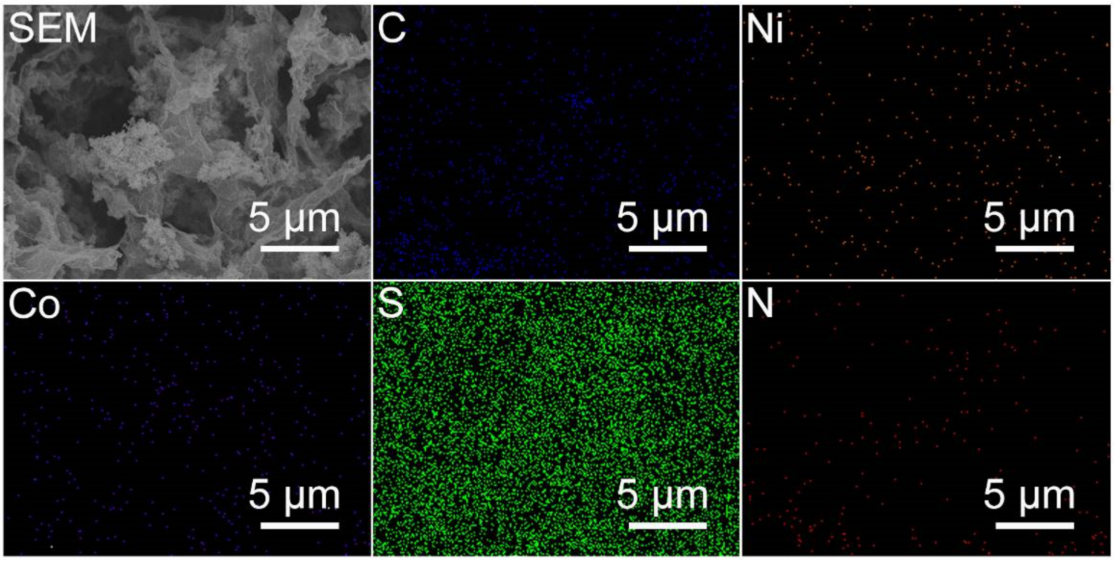

Figure. S4 Elemental mapping images of Co_2NiS_4/SNGA (C: light blue; Ni: orange; Co: dark blue; S: green and N: red)

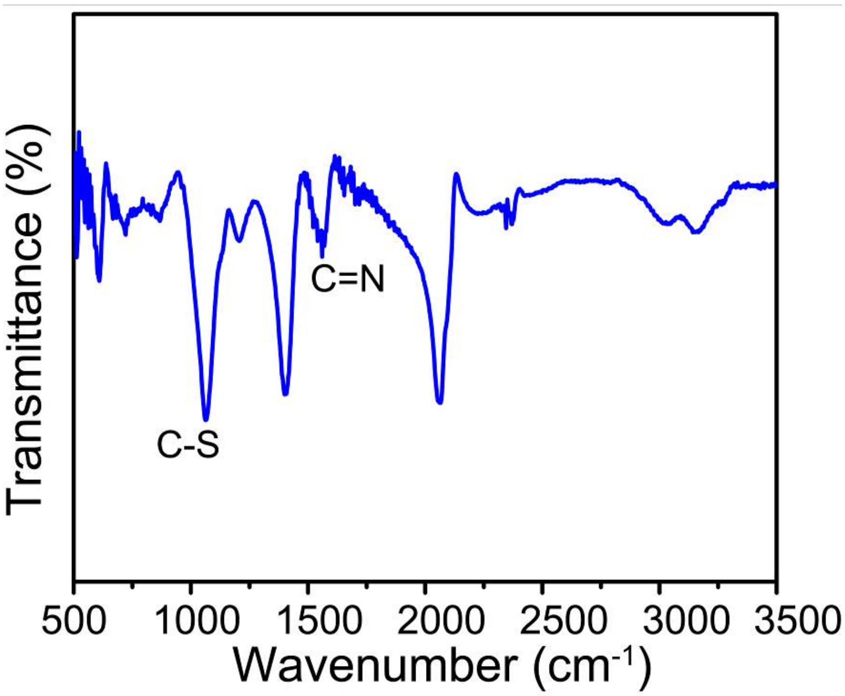

Figure. S5 FT-IR spectrum of $CoNi_2S_4$/SNGA

The C=N and C-S can be further evaluated by FTIR. The C=N streching at 1577 cm^{-1} and C-S stretching at 1068 cm^{-1} and the low intensity band at 601 cm^{-1} can be detected in the spectra.[1-3]

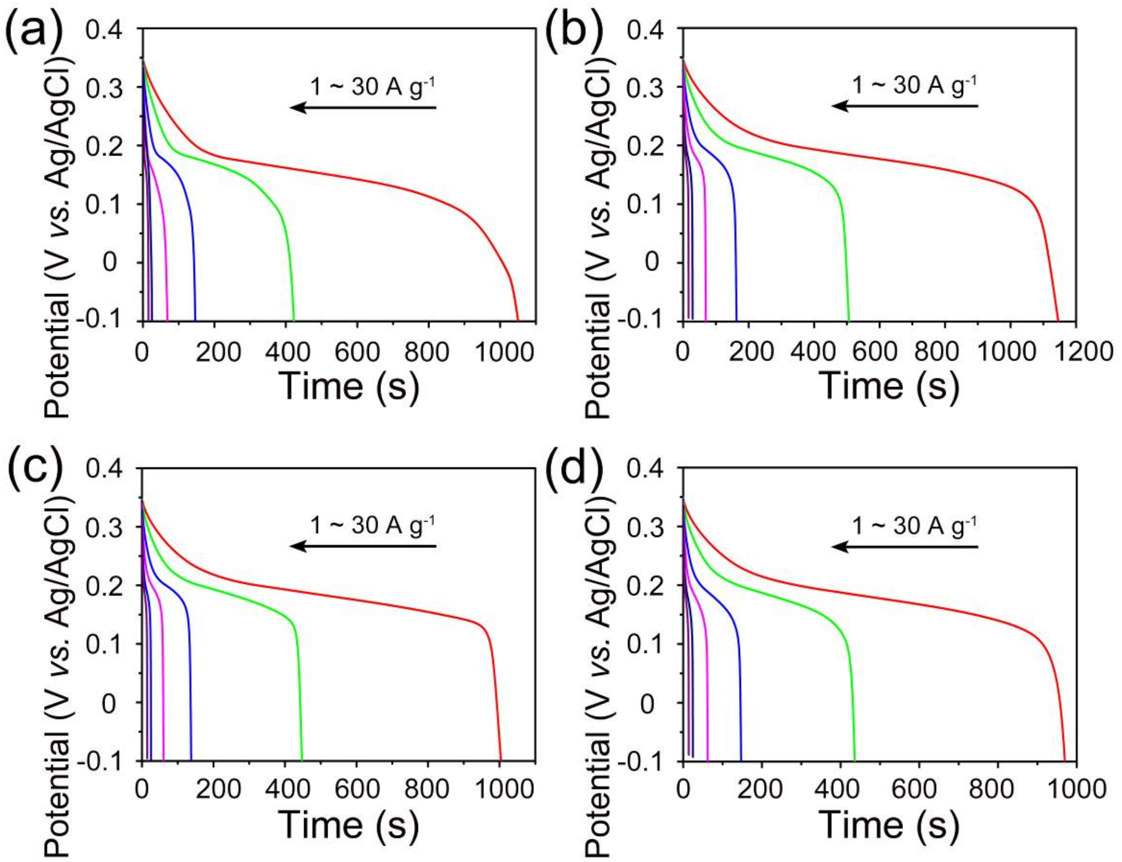

Figure. S6 Discharge curves of (a) $NiCo_2S_4$/SNGA, (b) $CoNi_2S_4$/SNGA, (c) Ni-S/SNGA, (d) Co-S/SNGA at the current densities of 1 ~ 30 A g^{-1} respectively.

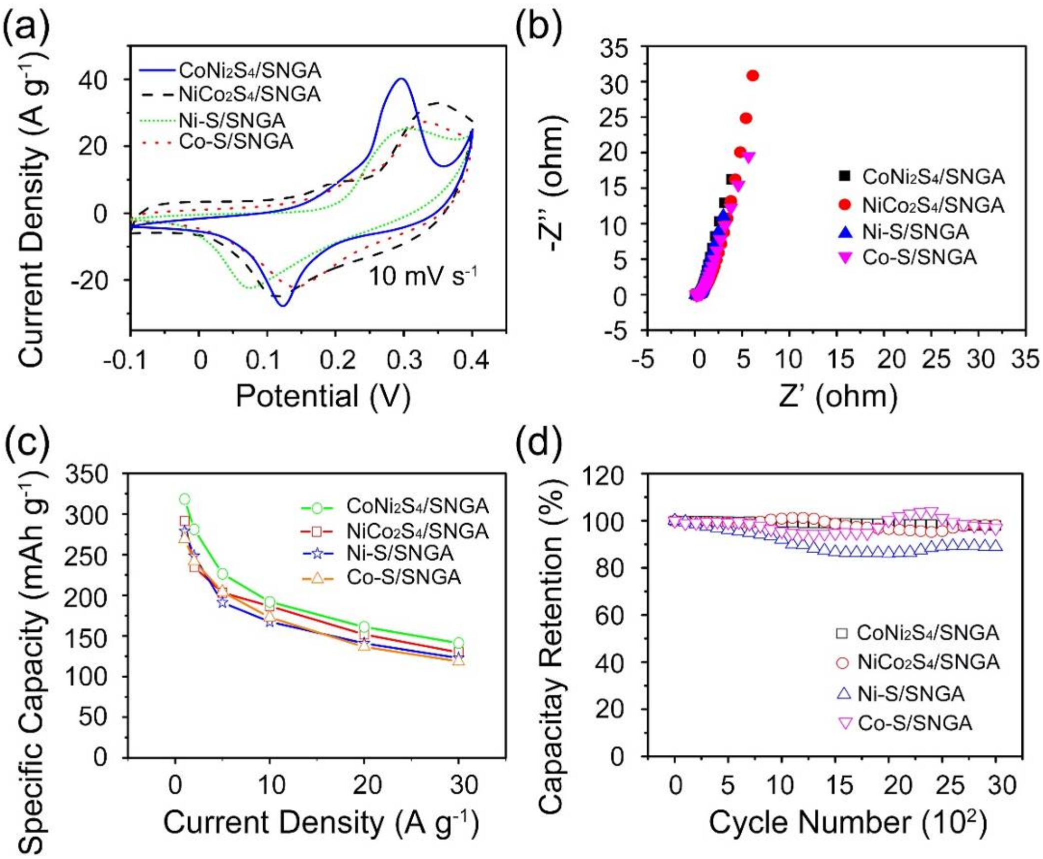

Figure. S7 (a) CV curves of $CoNi_2S_4$/SNGA, $NiCo_2S_4$/SNGA, Ni-S/SNGA and Co-S/SNGA at the scan rate of 10 mV s^{-1}, respectively; (b) Nyquist plot of the $CoNi_2S_4$/SNGA, $NiCo_2S_4$/SNGA, Ni-S/SNGA and Co-S/SNGA, respectively; (c) Rate capability of the $CoNi_2S_4$/SNGA, $NiCo_2S_4$/SNGA, Ni-S/SNGA and Co-S/SNGA at the current densities from 1~30 A g^{-1}, respectively. (d) Cycling performances of $CoNi_2S_4$/SNGA, $NiCo_2S_4$/SNGA, Ni-S/SNGA and Co-S/SNGA with the cycle numbers of 3000.

The calculations of the specific capacity were based on Figure. S4, the corresponding capacity retentions of the four kinds of samples were shown in Figure. S5 (c).

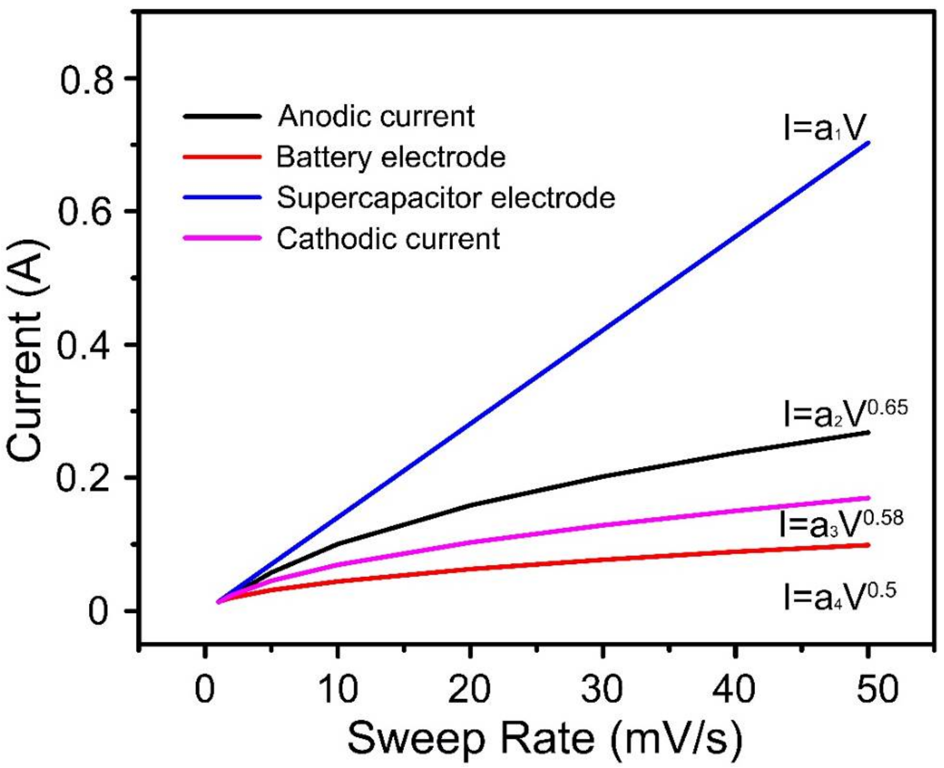

Figure. S8 The relation between peak current and the sweep rate from the CV curves of $CoNi_2S_4$/SNGA, ideal supercapacitor electrode and battery electrode, respectively.

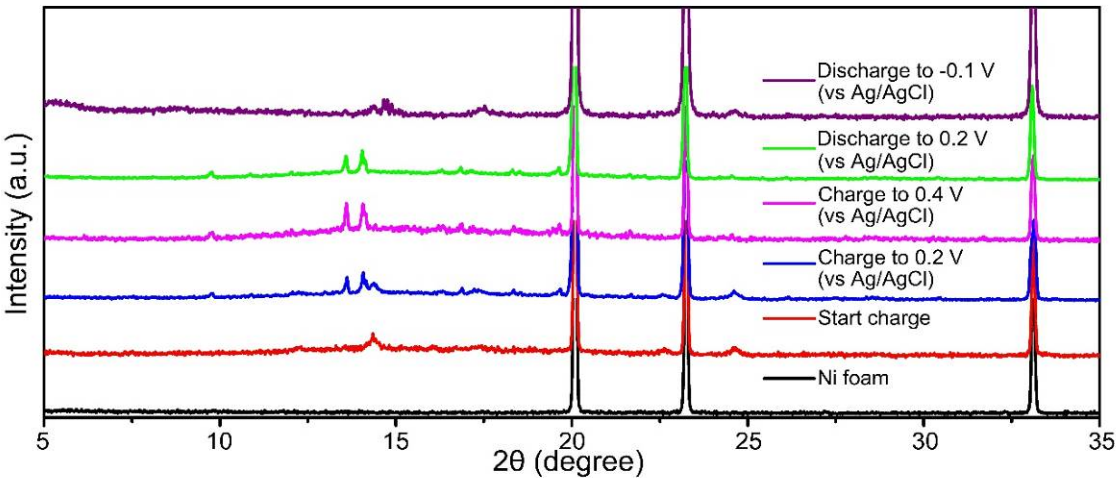

Figure. S9 *Ex-situ* XRD measurements of $CoNi_2S_4$/SNGA samples during different electrochemical steps.

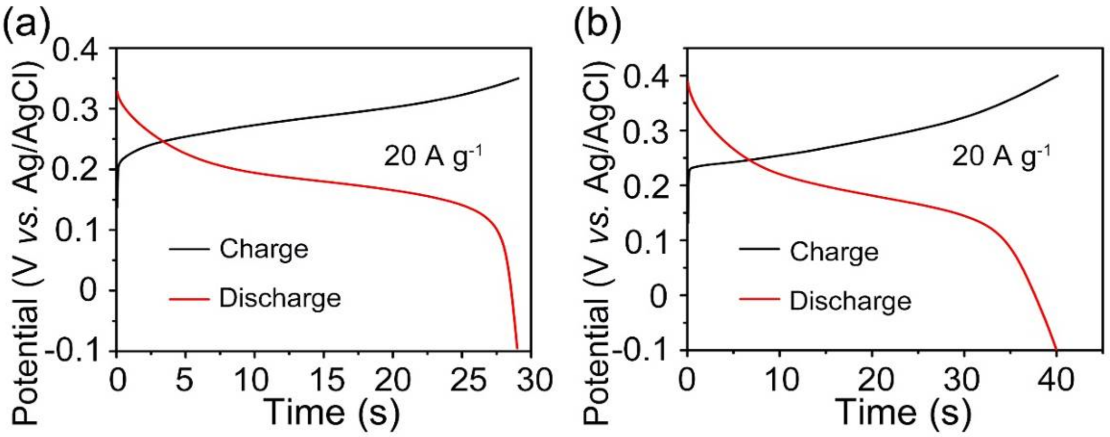

Figure. S10 GCD curves of $CoNi_2S_4$/SNGA at the current density of 20 A g^{-1} with different current window (a)-0.1~0.35 V (b) -0.1~0.4 V (*vs.* Ag/AgCl).

The calculation of the specific discharge capacity when the current density is 20 A g^{-1}:
When the voltage range is 0.45 V:
C=161.1 mAh g^{-1}
When the voltage range is 0.5 V:
C=222.1 mAh g^{-1}

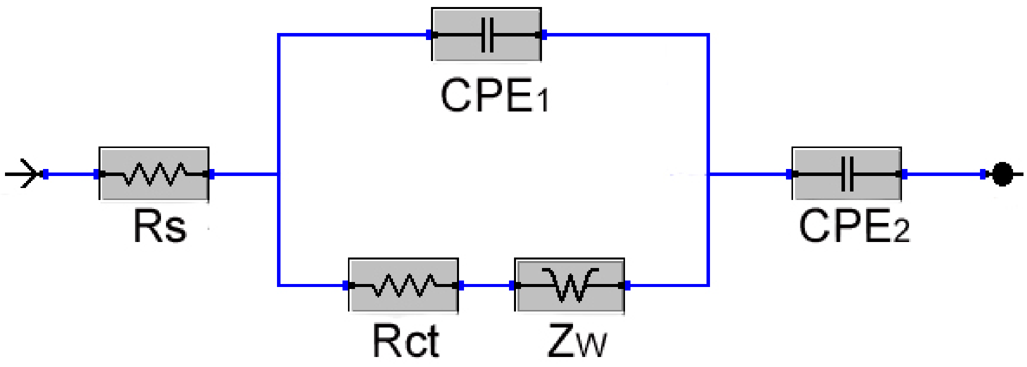

Figure. S11 Equivalent circuit of EIS for $CoNi_2S_4$/SNGA.

The parameter Rs is the inherent resistances of devices; CPE_1 and Rct are double-layer capacitance and charge transfer resistance, respectively; and Zw is the Warburg impedance related to the diffusion of OH^- ions into the bulk of the electrode. CPE_2 is caused by OH^- accumulating in the samples.

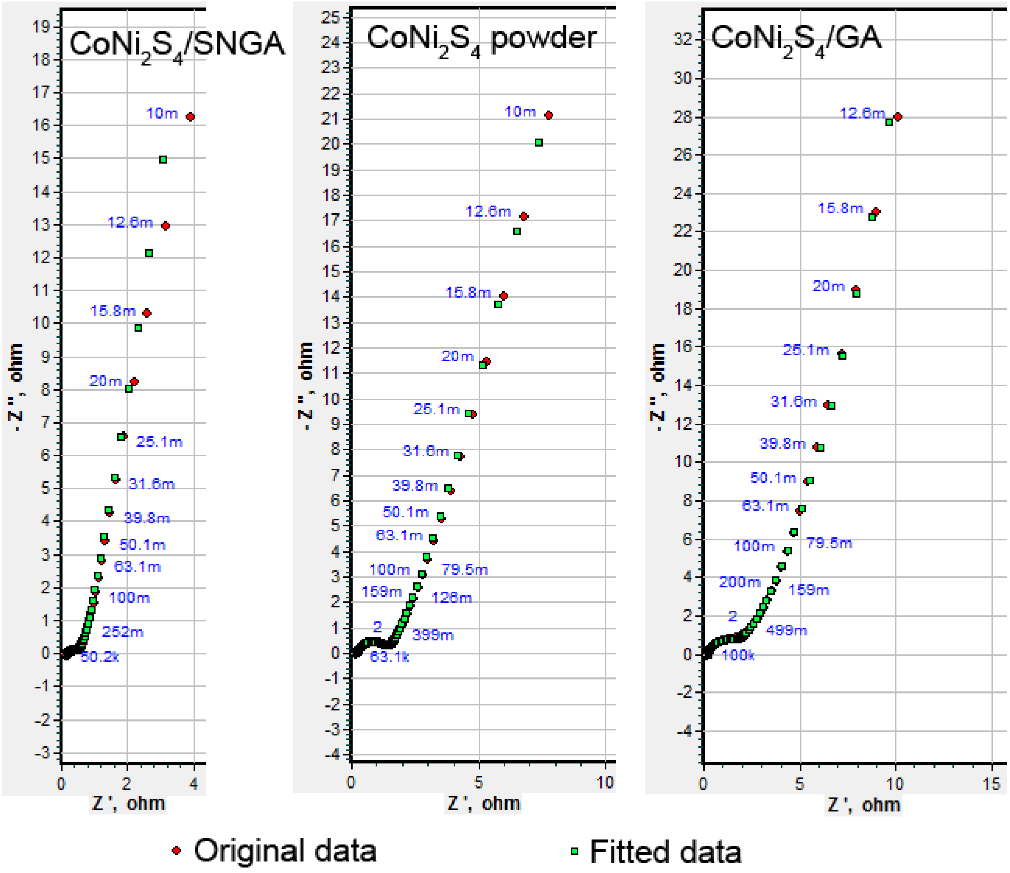

Figure. S12 Fitted EIS spectra for $CoNi_2S_4$/SNGA, $CoNi_2S_4$/GA and $CoNi_2S_4$ powder samples, respectively.

Table. S2 Comparison of rechargeable battery electrode performances of $CoNi_2S_4$/SNGA with some representative nickel/cobalt sulfide-based materials in the literature

nickel/cobalt sulfide-based materials	Specific capacity (mAh g^{-1})	Stability	Mass loading (mg cm^{-2})	References
$CoNi_2S_4$/SNGA	318.3 (1 A g^{-1})	~ 95.8% 10000 cycles	~ 2	Our work
Edge site-enriched nickel–cobalt sulfide into graphene frameworks	~ 194.8 (1 A g^{-1})	~ 90 % 8000 cycles	~ 3-3.5	[6]
CoS nanowire@$NiCo_2S_4$ nanosheet arrays	375.5 (10 mA cm^{-2})	~ 71.7 % 3000 cycles	~ 2	[7]
$CoNi_2S_4$ nanosheet arrays supported on nickel foams	363.3 (5 mA cm^{-2})	~ 78 % 3000 cycles	~ 2	[8]

Vapor-phase atomic layer deposition of Co_9S_8	205.6 (3 A g^{-1})	~ 94.4 % 2000 cycles	Not reported	[9]
$NiCo_2S_4$ nanosheets grown on nitrogen-doped carbon foams	205.2 (2 A g^{-1})	~ 90.4 % 2000 cycles	~ 2.3	[10]
Nickel cobalt sulfide ball-in-ball hollow spheres	158.3 (1 A g^{-1})	~ 87 % 2000 cycles	~ 5	[11]
$NiCo_2S_4$ nanoprisms	124.3 (1 A g^{-1})	~ 85.7 % 1500 cycles	~ 1	[12]
NiS_2 nanocube	77.2 (1.25 A g^{-1})	~ 93.4 % 3000 cycles	~ 2	[13]

*Specific capacity were calculated from specific capacitance and the voltage range in the references

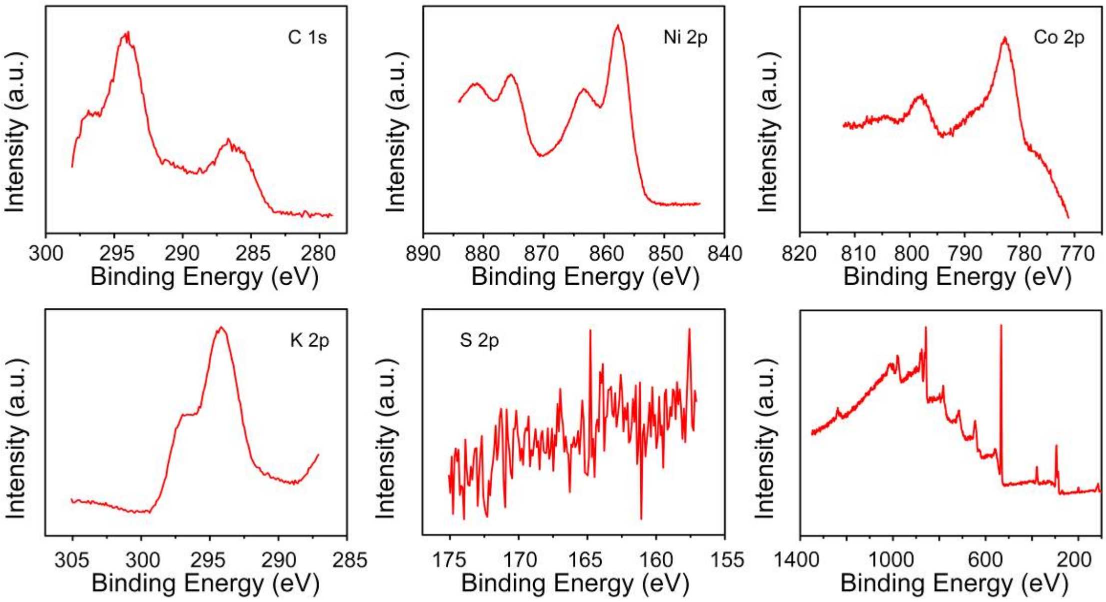

Figure. S13 XPS spectra of high-resolution of C 1s, Ni 2p, Co 2p, K 2p, S 2p and the survey spectra from $CoNi_2S_4$/GA samples after 3000 GCD cycles.

The peaks at ~ 284.8, ~ 286.3 and ~ 289.4 eV are due to the chemical state of C-C, C-O and C=O respectively. Two new peaks can be detected obviously at 293.0 and 295.8 eV after long-term cycling, which can be corresponded to the K 2p3/2 peak from the KOH electrolyte.

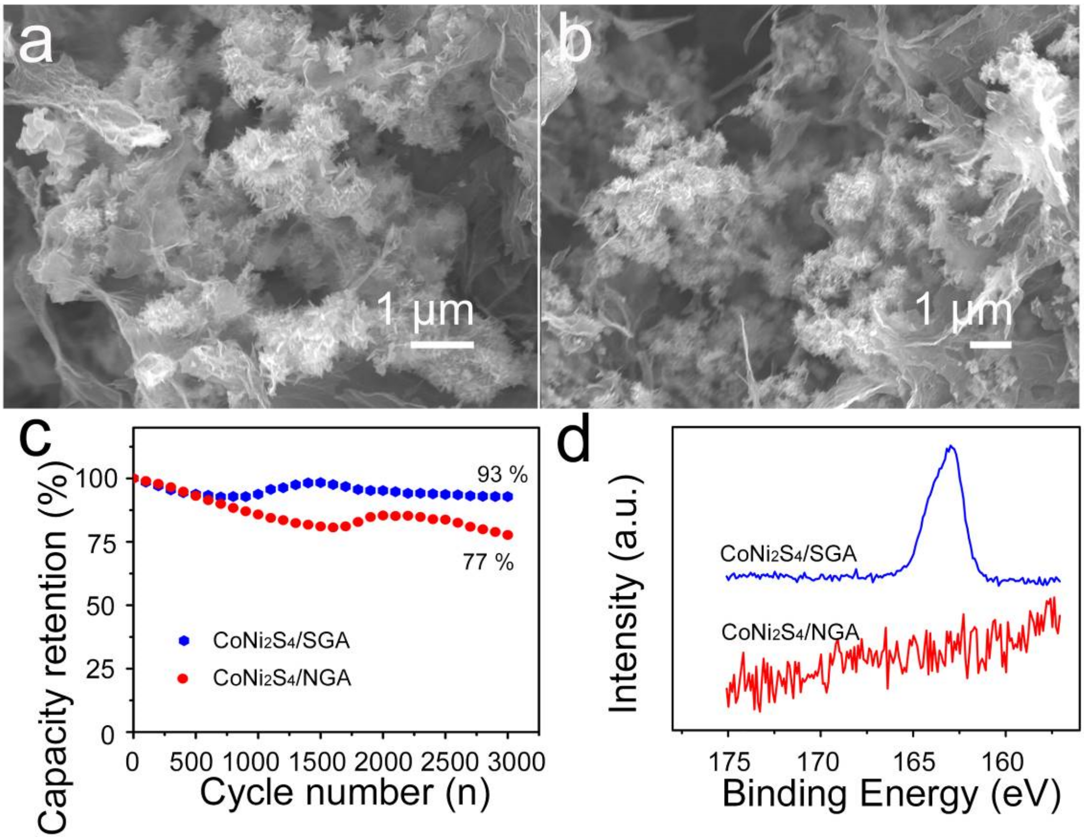

Figure. S14 (a, b) SEM images of $CoNi_2S_4$/NGA and $CoNi_2S_4$/SGA, respectively; (c) Stability test of the $CoNi_2S_4$/NGA and $CoNi_2S_4$/SGA for rechargeable alkaline battery electrodes in three electrode testing system; (d) S 2p spectra of $CoNi_2S_4$/NGA and $CoNi_2S_4$/SGA after cycling.

As can be seen from the SEM images, single doping of the graphene aerogels made almost no difference compared with co-doped graphene aerogels. The stability tests and the XPS data of S 2p proved the conclusions of the purpose of the N and S atoms.

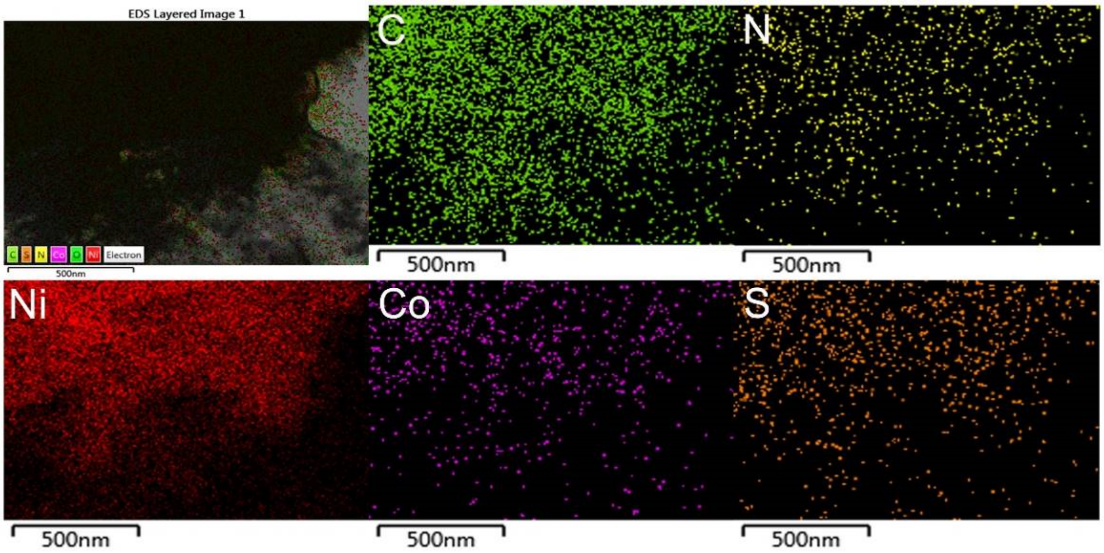

Figure. S15 TEM-EDS mapping images of the $CoNi_2S_4$/SNGA samples after 10000 GCD cycles.

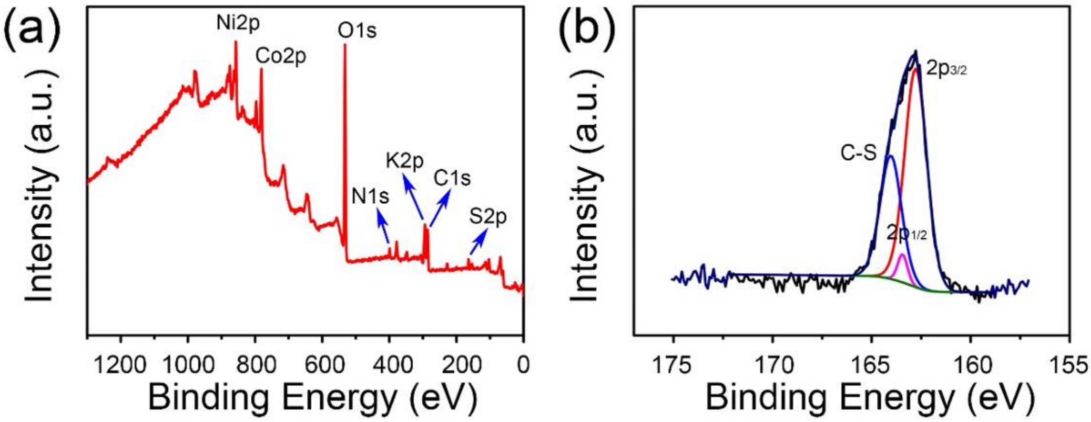

Figure. S16 XPS spectra of (a) survey and (b) high-resolution of S 2p from $CoNi_2S_4$/SNGA samples after 10000 GCD cycles.

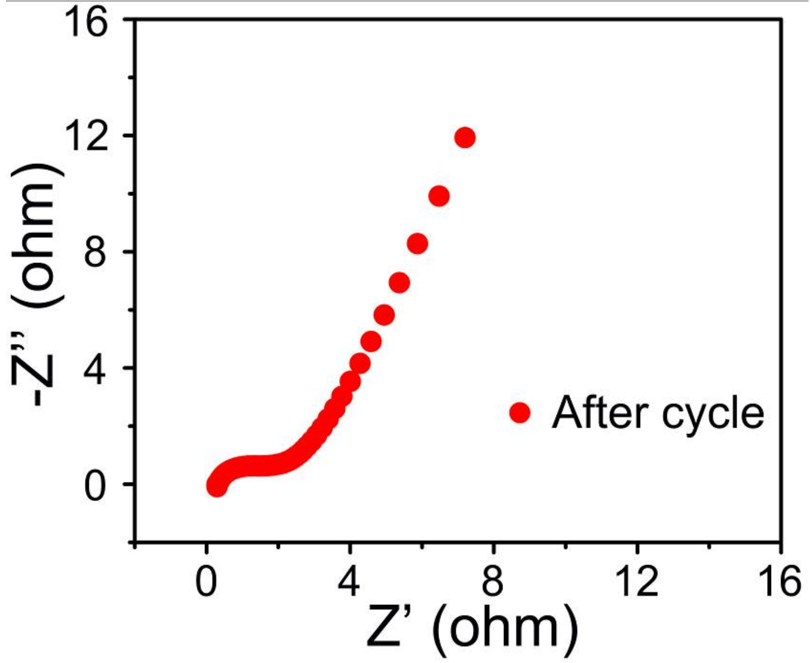

Figure. S17 EIS spectra of $CoNi_2S_4$/SNGA electrodes after 10000 GCD cycles.

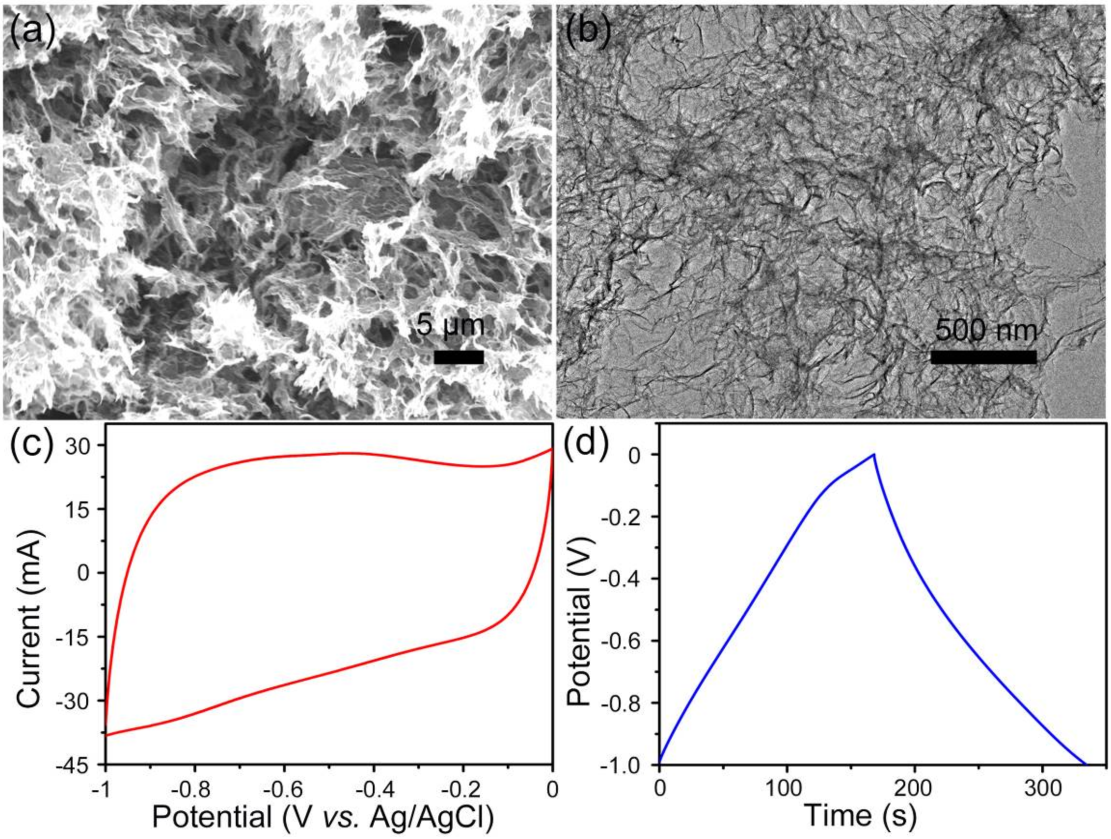

Figure. S18 (a), (b) SEM and TEM images of the SNGA, respectively; (c) CV curve of the SNGA electrode at the scan rate of 100 mV s^{-1}; (d) GCD curve of the SNGA at the current density of 1 A g^{-1}.

The calculation of the specific capacity of the SNGA is 46.2 mAh g^{-1} (specific capacitance: 166.4 F g^{-1}). According to the charge balance equation, the mass ratio of two electrodes is *ca.* 1: 7.

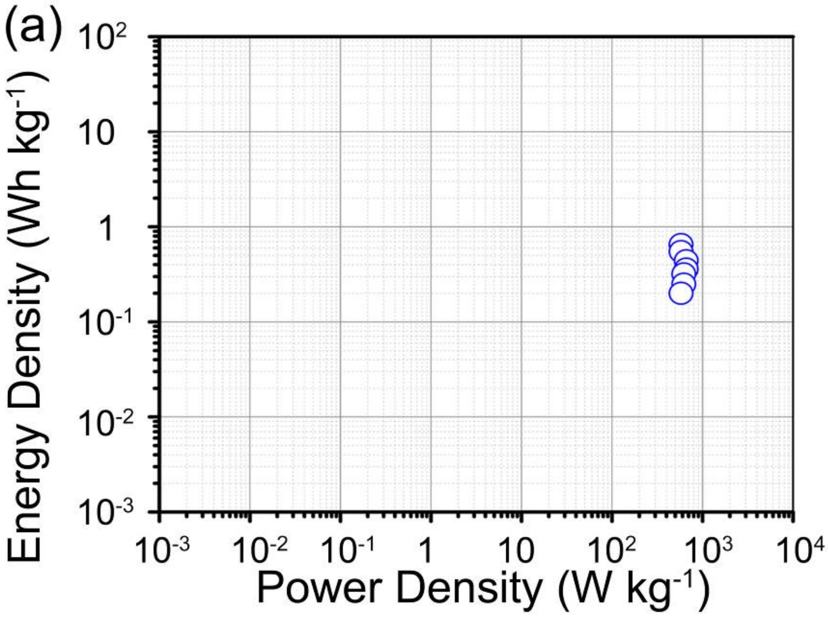

Figure. S19 Ragone plot of the device based on the total mass value.

Table. S3 Comparison of gel electrolytes and stability performances of solid-state devices from the literature with the solid-state battery in our work (SNGA//$CoNi_2S_4$/SNGA)

Solid-state devices	Gel electrolyte	Capacity retention	References
SNGA//$CoNi_2S_4$/SNGA AD	KOH/PVA gel	84 % 8000 cycles	Our work
Co_9S_8//Co_3O_4@RuO_2 AD	KOH/PVA gel	90.2 % 2000 cycles	[14]
SWCNTs//RuO_2 AD	H_3PO_4/PVA gel	~ 70 % 1000 cycles	[15]
WO_{3-x}/MoO_{3-x}//PANI/carbon fabric AD	H_3PO_4/PVA gel	75 % 10000 cycles	[16]
PPy/CNTs SD	H_3PO_4/PVA gel	73.8 % 10000 cycles	[17]

β-Ni(OH)$_2$/graphene SD	KOH/PVA gel	No detailed value 500 cycles	[18]
RuO$_2$/IL-CMG//IL-CMG AD	H_2SO_4/PVA gel	95 % 2000 cycles	[19]
NiCo$_2$O$_4$@PPy//AC AD	KOH/PVA gel	89.2 % 5000 cycles	[20]
PANI-ZIF-67 AD	H_2SO_4/PVA gel	80 % 2000 cycles	[21]

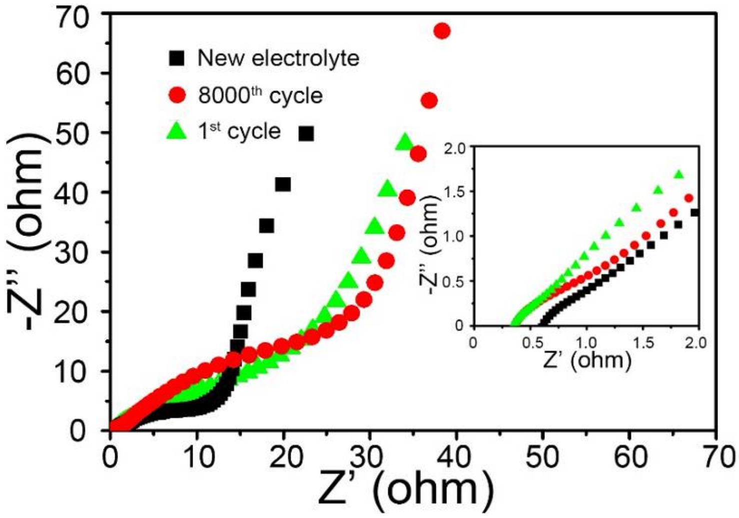

Figure. S20 EIS spectra before, after the cycling and when the new electrolyte dropping on the device.

In designing of new electrolyte dropping experiment, we wanted to verify the performances decline was mainly due to further water evaporation which inhibits the charge transfer and increases the Warburg resistance. With the addition of further electrolyte, it's obvious the inherent resistances of devices increased slightly which can be detected in the high frequency range.

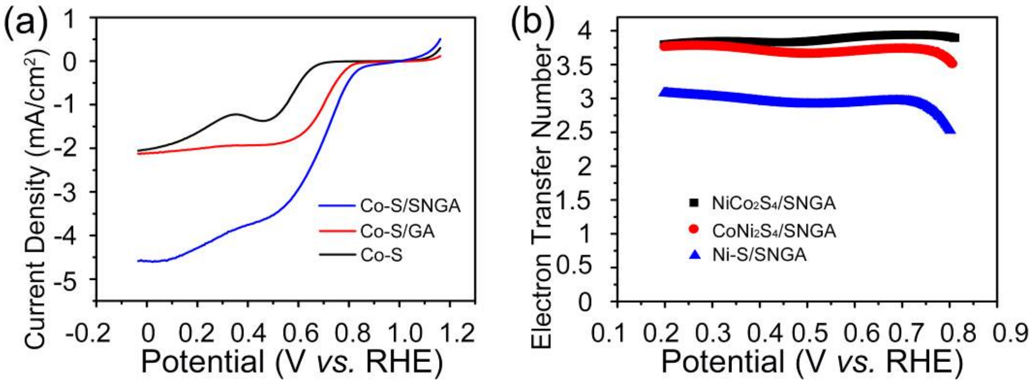

Figure. S21 (a) LSV spectra of the Co-S/SNGA, Co-S/GA and Co-S nanostructures at the rotation rate of 1600 rpm in O_2-saturated 0.1 M KOH, (b) electron transfer number of the $NiCo_2S_4$/SNGA, $CoNi_2S_4$/SNGA, Ni-S/SNGA calculated based on the RRDE value.

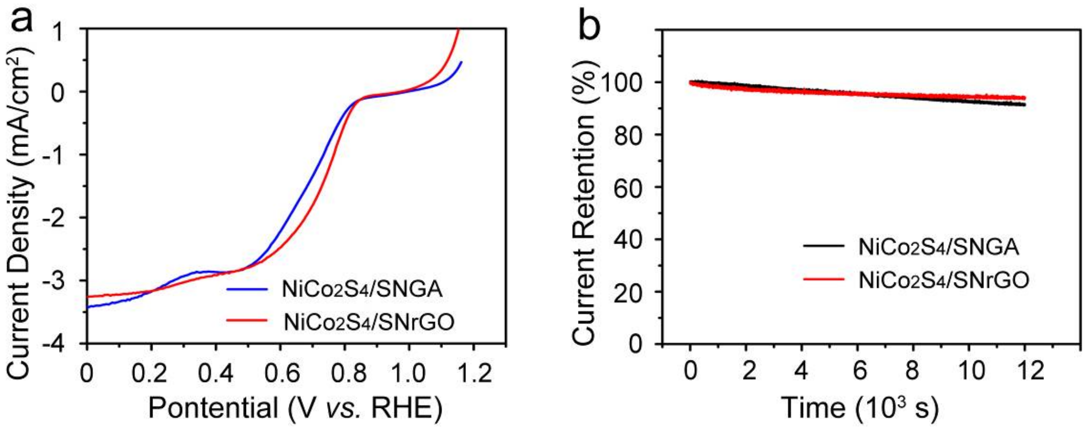

Figure. S22 (a) Rotating-disk electrode (RDE) measurements of $NiCo_2S_4$/SNGA and $NiCo_2S_4$/SNrGO in O_2-saturated 0.1 M KOH at 1600 rpm with a sweep rate of 10 mV s^{-1} (b) 12000 s of current-time chronoamperometric responses.

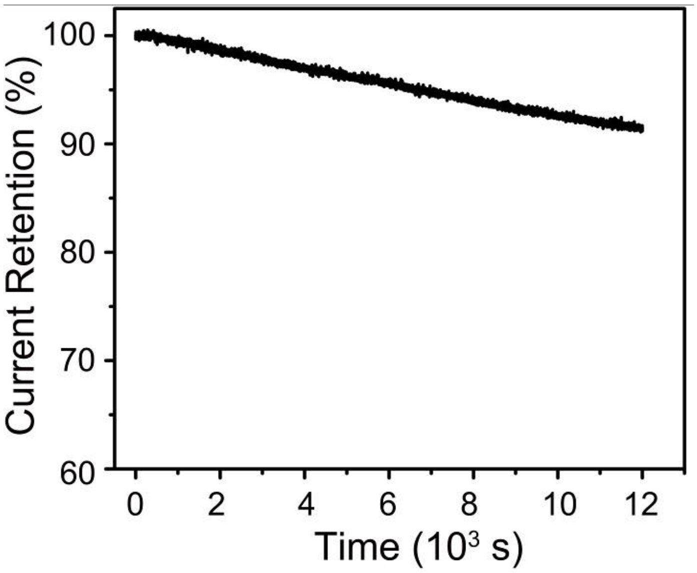

Figure. S23 Current−time chronoamperometric responses of $NiCo_2S_4$/SNGA at a rotation rate of 800 rpm for 12000 s in O_2-saturated 0.1 M KOH.

References

[1] Y. Xu, Z. Lin, X. Zhong, X. Huang, N. O. Weiss, Y. Huang, X. Duan, *Nat. Commun.*, **2014**, *5*, 4554.

[2] Y. Shao, H. Wang, Q. Zhang, Y. Li, *NPG Asia Mater.*, **2014**, *6*, e119.

[3]. F. Kanumfre, E. M. de Lima, G. Scheidt, P. I. B. Carneiro and N. D. Rosso, *J. Braz. Chem. Soc.*, **2010**, *21*, 800.

[4]. M. S. S. Babu, P.G. Krishna, K. H. Reddy and G. H. P. Chil, *Chem. Soc.*, **2009**, *54*, 339.

[5]. H. Liao, S. Chen and D. Liu, Macromolecules, **2009**, *42*, 6558.

[6] J. Yang, C. Yu, X. Fan, S. Liang, S. Li, H. Huang, Z. Ling, C. Hao, J. Qiu, *Energy Environ. Sci.*, **2016**, DOI: 10.1039/c5ee03633j.

[7] W. Zeng, G. Zhang, X. Wu, K. Zhang, H. Zhang, S. Hou, C. Li, T. Wang, H. Duan, *J. Mater. Chem. A*, **2015**, *3*, 24033.

[8] W. Hu, R. Chen, W. Xie, L. Zou, N. Qin, D. Bao, *ACS Appl. Mater. Interfaces*, **2014**, *6*, 19318.

[9] H. Li, Y. Gao, Y. Shao, Y. Su, X. Wang, *Nano Lett.*, **2015**, *15*, 6689.

[10] L. Shen, J. Wang, G. Xu, H. Li, H. Dou, X. Zhang, *Adv. Energy Mater.*, **2015**, *5*, 1400977.

[11] L. Shen, L. Yu, H. B. Wu, X. Y. Yu, X. Zhang, X. W. Lou, *Nat. Commun.*, **2015**, *6*, 6694.

[12] L. Yu, L. Zhang, H. B. Wu, X. W. Lou, *Angew. Chem. Int. Ed.*, **2014**, *53*, 3711.

[13] H. Pang, C. Z. Wei, X. X. Li, G. C. Li, Y. H. Ma, S. J. Li, J. Chen, J. S. Zhang, *Sci. Rep.*, **2014**, *4*, 3577.

[14] J. Xu, Q. Wang, X. Wang, Q. Xiang, B. Liang, D. Chen, G. Shen, *ACS Nano*, **2013**, *7*, 5453.

[15] P. Chen, H. Chen, J. Qiu, C. Zhou, *Nano Res.*, **2010**, *3*, 594.

[16] X. Xiao, T. Ding, L. Yuan, Y. Shen, Q. Zhong, X. Zhang, Y. Cao, B. Hu, T. Zhai, L. Gong, J. Chen, Y. Tong, J. Zhou, Z. L. Wang, *Adv. Energy Mater.*, **2012**, *2*, 1328.

[17] H. Lin, L. Li., J. Ren, Z. Cai, L. Qiu, Z. Yang, H. Peng, *Sci. Rep.*, **2013**, *3*, 1353.

[18] J. Xie, X. Sun, N. Zhang, K. Xu, M. Zhou, X. Yie, *Nano Energy*, **2013**, *2*, 65.

[19] B. G. Choi, S. Chang, H. Kang, C. P. Park, H. J. Kim, W. H. Hong, S. Lee, Y. S. Huh, *Nanoscale*, **2012**, *4*, 4983.

[20] D. Kong, W. Ren, C. Cheng, Y. Wang, Z. Huang, H. Y. Yang, *ACS Appl. Mater. Interfaces*, **2015**, *7*, 21334.

[21] L. Wang, X. Feng, L. Ren, Q. Piao, J. Zhong, Y. Wang, H. Li, Y. Chen, B. Wang, *J. Am. Chem. Soc.*, **2015**, *137*, 4920.
